# Recent Advances in Preparation, Modification, and Application of Free-Standing and Flow-Through Anodic TiO_2_ Nanotube Membranes

**DOI:** 10.3390/molecules29235638

**Published:** 2024-11-28

**Authors:** Ewelina Szaniawska-Białas, Anna Brudzisz, Amara Nasir, Ewa Wierzbicka

**Affiliations:** 1 Department of Functional Materials and Hydrogen Technology, Faculty of Advanced Technologies and Chemistry, Military University of Technology, 2 Kaliskiego Street, 00908 Warsaw, Poland; ewelina.szaniawska@wat.edu.pl (E.S.-B.); brudziszanna@gmail.com (A.B.); 2 Department of Physics, The University of Lahore, Lahore 53700, Pakistan; amaranasir.an@gmail.com

**Keywords:** anodizing, titania nanotubes membranes, detachment, hydrogen evolution, Li-S batteries

## Abstract

Free-standing and flow-through anodic TiO_2_ nanotube (TNT) membranes are gaining attention due to their unique synergy of properties and morphology, making them valuable in diverse research areas such as (photo)catalysis, energy conversion, environmental purification, sensors, and the biomedical field. The well-organized TiO_2_ nanotubes can be efficiently and cost-effectively produced through anodizing, while further utility of this material can be achieved by creating detached and flow-through membranes. This article reviews the latest advancements in the preparation, modification, and application of free-standing and flow-through anodic TiO_2_ nanotubes. It offers a comprehensive discussion of the factors influencing the morphology of the oxide and the potential mechanisms behind the electrochemical formation of TiO_2_ nanotubes. It examines methods for detachment and opening the bottom ends to prepare free-standing and flow-through TNT membranes and posttreatment strategies tailored to different applications. The article also provides an overview of recent applications of these materials in various fields, including hydrogen production, fuel and solar cells, batteries, pollutant diffusion and degradation, biomedical applications, micromotors, and electrochromic devices.

## 1. Introduction

This article provides an overview of the latest advancements in the preparation, modification, and application of free-(self-)standing and flow-through titania anodic nanotube (TNT) membranes. The work is focused on the recent progress made in this field, particularly on the last decade’s achievements.

Titanium dioxide, as a wide band gap, *n-type* oxide semiconductor, has gained extensive research interest, mainly due to the broad perspective of its utilization in multiple technologies, including photocatalysis (PC), hydrogen production, dye-synthesized solar cells (DSSC), and biomedical applications [1,2]. Significant progress has been achieved when nanostructures of TiO_2_ in various forms, such as nanopowders, nanotubes/nanoporous layers attached to the metal surface, have been developed. Their functionality and unique properties are strongly improved by the quantum confinement effect and a great surface-to-volume ratio [3].

TiO_2_ nanotubes (TNTs) can be synthesized using various methods, including template-based synthesis, hydrothermal treatments, electrospinning, or electrochemical anodization [3,4]. Among these, the anodic oxidation of titanium (Ti) foils stands out for its utility, as it allows for the development of self-organized TiO_2_ nanotubes with controllable morphology through a relatively simple and low-cost approach.

Zwilling et al. already reported in 1999 that anodizing titanium substrate in a fluoride-containing electrolyte leads to the formation of ordered TiO_2_ nanotube arrays (TNTa) [5]. Since then, one-dimensional (1D) titanium dioxide nanotubes have become the subject of numerous studies in diverse research areas. The TNT arrays possess a well-ordered structure, which provides an extra-large surface area, good electrical conductivity, and efficient separation of photoinduced charge carriers—much better than reported for dense nanoparticle (NP) layers [6,7,8]. After anodizing, the prepared TNTs are well attached to a titanium substrate, which is strongly beneficial in some applications, e.g., they show good adhesion to Ti back-contact in photoanodes. However, the presence of titanium foil might have some drawbacks in other applications, such as a lack of transparency or a closed nanopore structure (lack of flow-through). For this reason, multiple new flow-through TNT membrane preparation strategies have been developed, including chemical, electrochemical, mechanical, or combinatory methods, which will be discussed in detail in this overview.

The membrane morphology of TiO_2_ nanotubes opens a new perspective for the material’s applications. Similar to titania nanotubes, it shows thermal, chemical, and corrosion resistance, non-toxicity, and low cost of production. Except for using the membrane “as is”, it can also be attached to any foreign substrate/support material, e.g., transparent foils or glass, providing a suitable solution for energy conversion applications. The unique material features, such as nanoporosity, flow-through channels, and semiconductor nature with light absorption in the solar range, make it a compelling candidate for energy conversion, degradation, and filtration applications, especially after targeted material modifications.

First, for keeping membrane integrity after the detachment process and bottom opening, if a flow-through morphology is required, suitable anodizing conditions must be selected to form a stable TNT array (e.g., proper thickness, array compactness, and limiting surface cracking). Factors to consider include the type of electrolyte (organic vs. inorganic) [9,10,11]; the concentration of fluorides [12,13,14,15,16]; the duration of the process [11,14,17,18,19,20,21,22,23,24,25,26,27,28]; applied current/voltage [10,11,16,29,30,31,32]; water content [10,16,29,30,33,34]; and more. The second challenge in forming TNT membranes is their detachment from the Ti substrate after anodizing. So far, multiple methods of TNT membrane preparation, with well-described mechanisms of detachment, have been offered in the literature, e.g., chemical separation, mechanical delamination, electrochemical treatment, self-detachment, plasma etching, or a combination of these methods. Frequently, in the sequence of the detachment steps, the conversion of amorphous titania to crystalline structure is included to stabilize the membrane and make it resistant to further treatments. Finally, it must be noted that if the flow-through membrane morphology is required, the nanotube bottom opening might be achieved either simultaneously with the detachment process or in the following step by chemical etching [17,26,35,36,37], ion milling with Ar^+^ bombardment [38,39,40,41], ion beam or plasma etching [42,43].

A retrospective analysis of the research on TNT membranes over the past decade demonstrates notable advancements in membrane modification strategies. An obvious benefit of such a system comes from the absence of Ti substrate, which enables flow-through system formation, as well as site-selective modifications [17]. Except for this fundamental benefit of membrane architecture, traditional approaches such as composite materials formation (with noble metals [12,13,17,28,39,41,44,45,46,47,48,49] and/or non-metals [39,40,48,50]; non-metallic dopants [27,38]; quantum dots [36,51,52,53,54,55,56]; heterostructures with other semiconductors [18,25]), also find application here. The material has been widely researched in the direction of hydrogen generation [6,12,13,17,18,28,44,45]; fuel [46,57] and solar cells [14,15,19,20,21,22,23,24,25,26,27,39,40,41,42,47,49,51,52,53,54,58,59,60,61,62,63,64,65,66,67,68,69,70,71,72,73,74,75,76,77,78,79,80,81,82]; Li-S batteries [83]; organic pollutant diffusion and degradation [37,84,85,86,87,88,89,90]; biofiltration and sensing [50,91,92,93,94,95]; micromotors [96]; and electrochromic devices [97,98,99].

It has emerged from the literature review that there is a crucial need for a comprehensive investigation of the optimization of electrochemical anodizing parameters (electrolyte composition, temperature, applied voltage, etc.) for obtaining a crack-free TiO_2_ nanotube membrane and tailoring its structural and functional properties. This review summarizes the recent reports on the approaches to membrane preparations, including anodizing conditions, and strategies for TNT layer detachment and nanotube bottom opening to form flow-through channels. Moreover, multiple approaches developed to enhance the material properties and improve overall activity in the context of varied specific applications have also been discussed.

## 2. Self-Organized Titanium Dioxide Nanotubes (TNTs) Membrane Preparation

### 2.1. Electrochemical Anodizing Method

Electrochemical oxidation can be carried out using aqueous or organic-based electrolytes [33,100,101,102,103]. An anodic oxide film forms a barrier-type layer in most aqueous electrolytes (such as sulphuric, phosphoric, or acetic acid). However, it was found that anions in electrolytes such as fluoride, perchlorate, chloride, or bromide can act as etching agents [11,33,102,103], facilitating the direct chemical ejection/dissolution of metal ions during anodizing. This results in transitions from the barrier layer into a nanoporous/nanotubular oxide structure. The process is typically performed in the two-electrode cell configuration, where the titanium substrate serves as an anode and a platinum electrode works as a cathode. When voltage is applied across the electrodes, under selected anodizing conditions, a current flow induces titanium oxide formation on the titanium substrate [10,104]. Anodizing parameters are highly controllable and easily adjustable. Therefore, the method is commonly utilized to form different morphologies of TiO_2_ nanotube arrays [33,105]. As noted previously, the nanotube morphology strongly depends on the electrolyte’s chemical nature, but also stirring, temperature, anodizing parameters such as voltage or current density applied, and process duration [10,11,16,31,32].

In organic electrolytes with high viscosity and low water content, smoother and longer nanotubes without ripples can be obtained. The water content in the electrolyte influences the splitting of tube walls, affecting the compactness of the nanotube array [10,16,29,30,33,34]. For instance, using ethylene glycol (EG) with 0.3 wt% NH_4_F and 2 vol% H_2_O can result in nanotubes up to 360 μm in length with a high growth rate of 15 µm/h [9]. Based on the experimental data collected over the past decade on the nanotube titania membranes, the thickness ranges from 1.5 µm to 110 µm (refer to Table 1, Table 2 and Table 3 in the following sections). It should be noted that nanotubes in the micrometer range can be fabricated using fluoride salt as an F^−^-source [29]. In particular, when anodizing Ti foil in EG with 0.15–0.5 wt% NH_4_F and 3 vol% H_2_O at 60 V for 1 h, nanotubes length can range from 15 μm to 23.8 μm [12,13,14,15,23] (see Table 1, Table 2 and Table 3). The disadvantage of utilizing hydrofluoric acid (HF) electrolytes is limiting the maximum thickness of prepared TiO_2_ nanotubes to only a few hundred nanometers due to the high rate of chemical dissolution of TNT by fluorine ions [30]. In addition, the increasing water content in electrolytes accelerates chemical dissolution, increasing the nanotube diameter. Nanotubes with an inner diameter ranging from ∼30 nm to ∼150 nm can be formed by applying voltages between 40 and 80 V in ethylene glycol electrolytes with 0–3 wt% of H_2_O and 0.15−0.8 wt% F^−^ (see Table 1, Table 2 and Table 3). According to Raja et al., a minimum of 0.18 wt% water content in organic electrolytes is required to obtain a well-ordered TiO_2_ nanotube array, while water concentration higher than 0.5 wt% causes the formation of ridges [34].

The physical parameters of the anodizing are critical for TNT features and architecture, in addition to the electrolyte composition. There is a direct relationship between anodizing voltage and resulting TNT morphology. As the voltage increases, the diameter and length of the tube also increase. Wang et al. [32] indicated that the pore diameter and membrane thickness changed with the anodizing voltage in the range of 20–80 V from 30 nm to 200 nm and 5 µm to 32 µm, respectively. The length of the nanotubes is further controlled by the duration of anodizing at a certain voltage. For instance, when the anodizing time was increased from 4 to 10 h, the corresponding lengths of TNTs were 38 µm and 110 µm, respectively [18,28]. Additionally, under specific measurement conditions (i.e., electrolyte type and temperature), the relation between tube diameter and the applied voltage is linear [11,29,30,33,106]. It should be noted that at high voltage, electrochemical oxidation of the Ti substrate in organic electrolytes induces Schottky breakdown, leading to electrolyte decomposition and the generation of a carbon-rich contamination layer. According to the data presented in Table 1, Table 2 and Table 3, nanotube membranes are typically produced at 50 V [24,36,44,54,60,62,107] or 60 V [12,13,14,15,18,19,20,21,22,23,25,26,27,28,39,40,41,42,49,52,53,55,56,68,69,70,71,73]. These voltage values appear to strike a balance, promoting nanotube growth while preventing defect formation or undesirable properties [16,32]. Thus, anodizing voltages higher than 60 V can result in faster growth rates but also the formation of larger defects, irregularities, or breakdown events in the nanotube structure [16,32].

Moreover, to achieve a more organized distribution of pores/nanotubes, researchers have shown interest in pre-anodizing titanium before growing the desired nanotubes. Therefore, the initial TNT array was effectively removed through ultrasonication, exposing the surface dimples on the Ti substrate [14,17,18,19,92]. These small depressions serve as preferential centers for uniform nanotube growth during the subsequent anodizing (see Table 1, Table 2 and Table 3).

Frequently, before the anodizing process, the titanium substrate undergoes cleaning to eliminate surface impurities and polishing to reduce surface roughness [108] to improve final structure ordering. TiO_2_ nanotubes can be fabricated through single- [12,13,28,32,36,42,44,45,50,60,69,70,72,78,91,109] or multi-step [14,17,18,19,20,21,22,23,24,25,26,27,84,85,92,110,111] anodizing. TNTs obtained through a one-step anodizing process display imperfect lateral ordering, while those prepared by multi-step anodizing are grown on a pre-anodized Ti substrate and demonstrate a more close-packed hexagonal arrangement [92]. An approach Yanagishita et al. [112] proposed involves pre-anodizing the Ti substrate, followed by two additional anodizing steps to achieve ideally ordered flow-through TiO_2_ nanotube membranes. In this technique, the hole period can be precisely controlled and kept at 200 nm by the authors. Importantly, after detaching the prepared membrane, the Ti substrate can be repeatedly re-used to form new well-ordered TNT membranes [112].

After the nanotube array is grown on the substrate, it may undergo post-treatment processes, depending on its intended application [113,114]. Most frequently, it is annealed to form a crystal structure, as the as-anodized TNTa are amorphous. For TNT membranes, it must be considered that the annealing conditions determine the crystal structure and, therefore, semiconductor properties (an important factor in many applications such as photocatalysis). Conversely, it might cause surface cracking and destabilization if overheated. Therefore, in the literature, the annealing conditions for TNT membranes do not exceed 650 °C, as temperatures beyond this can lead to the degradation of the nanostructure morphology [33,115].

Anodizing is a relatively undemanding technique, providing very precise control over the titania nanotube structure and morphology [10,16]. Therefore, it is invaluable as a powerful synthesis method for TNTs and their self-standing membranes, which can be successfully utilized in multiple scientific and technological fields and applications.

#### TNTs Formation Mechanism

**Figure 1:**
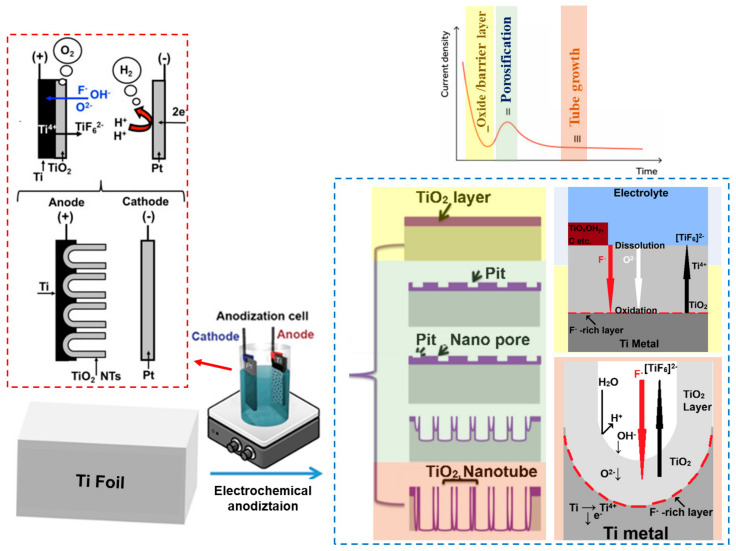
Electrochemical oxidation process for the growth of TiO_2_ nanotubes. The colored regions on the left represent the different stages of the anodizing process. Adapted with permission from [102]. Copyright © 2013, Elsevier. Adapted with permission from [119]. Copyright © 2017, Springer Nature.

The mechanism underlying nanopore formation through anodizing remains the subject of debate despite the numerous studies in this field. Among the proposed theories, particularly distinguished models include field-assisted dissolution (FAD)/ejection (FAE), plastic flow, ionic and electronic current theory, and oxygen bubble mold effect, and self-organized theories [116,117,118]. The most conventional but simplistic growth model for TiO_2_ nanotube formation follows a field-assisted dissolution (FAD) and oxidation process [3]. Figure 1 provides a general scheme of an electrochemical anodizing process in F^−^containing solution, which includes the reactions involved in the growth process of TNTs (inset on the left-hand side), together with a graphical dependence of current density on anodizing time [33,102,119]. Initially (*stage I*), when a voltage is applied to the titanium substrate immersed in the electrolyte solution, an electric field triggers anodic oxidation of the titanium surface, releasing electrons and Ti^4+^ ions. Titanium cations interact with oxygen (O^2−^) and hydroxide (OH^−^) anions from electrolytes, forming a hydrated anodic oxide layer. In the plot of current density vs. time, *stage I* is indicated by a significant drop in the recorded current (Figure 1). In most fluorine-free anodizing solutions, the resulting anode oxide layer is of a barrier type. The anodizing process continues until the layer reaches a critical thickness, at which point the applied electric field’s ion migration is prevented. The presence of fluorine ions is found to be crucial in pore formation at the interface of the anodic oxide layer/electrolyte. Therefore, in *stage II*, a slight increase in current density was attributed to the formation of small pores or pits that originate from the etching of the anodic oxide layer by F^−^ ions. These pores or pits serve as nucleation sites for the growth of nanotubes. As the anodizing process continues, these pits grow deeper in the TiO_2_ layer, *stage III*. The plateau observed in the current density-time curve indicates a stable growth of pores [33,102,118]. Field-assisted dissolution theory assumes that oxide formation at the anode surface competes with the oxide dissolution during anodizing. However, if the electric field and electrolyte remain unchanged, the FAD reaction should continue indefinitely, leading to continuous nanotube growth, which contradicts experimental results [118].

The theory of the ionic and electronic current and oxygen bubble mold effect is similar to the FAD theory, but only during the *stage I* of anodizing when the oxides form simultaneously at both the oxide layer/electrolyte and the oxide layer/metal interfaces [102,118,120]. In the FAD theory, once a porous structure forms, oxide growth only occurs at the metal/oxide layer interface, while dissolution occurs at the electrolyte/oxide layer interface. In contrast, in the oxygen bubble mold effect theory, as the thickness of the oxide layer increases, the migration of ions through the oxide layer becomes more challenging, and the barrier layer, being in direct contact with the electrolyte, is partially transformed into an anion contaminated layer (ACL). The growth of the oxide layer is attributed to ionic current. Once the barrier layer reaches the critical thickness, an increased amount of electronic current is generated, and the process enters *stage II*. At this stage, oxygen bubbles are produced and become trapped between the ACL and the barrier layer and cannot be released to the electrolyte due to factors such as ALC and electrolyte pressure. These trapped oxygen bubbles slowly expand, guiding the creation of porous anodic oxides (PAO) resembling mold. After breaking through the layer contaminated with anions, oxygen bubbles are eventually released from the surface, and the electrolyte reaches the bottom of the nanotubes. This transforms the nanotube embryos into nanotubes (*stage III*) [118]. This step involves achieving a balance between the rate of oxygen release and the rate of oxide growth. In addition, the theory of the oxygen bubble mold effect aligns with the self-organized theory regarding the impact of oxygen bubble evolution on pore morphology [118].

In comparison with the FAD and FAE theories described earlier, the self-organization theory draws attention to the key influence of anodizing conditions, such as voltage, solubility, field effects, or stress, on the porous anodic oxide formation [33,102,118,121]. The oxide layer grows during anodizing through ionic migration, controlled by a high electric field (*stage I*). As the thickness of the oxide layer increases, the electric field across the layer decreases. This defines a self-limiting process that is directly related to the applied voltage. Achieving prolonged anodic oxide growth involves establishing an equilibrium between the formation and dissolution of oxide, which allows for further evaluation of a nanotubular/porous structure. Thus, the solubility of metal oxide in the electrolyte is a key factor for self-organized PAO formation. The abovementioned might be obtained by fluorides, which can either chemically dissolve the oxide or complex Ti^4+^ ions as [TiF_6_]^2−^ (*stage II*). The reaction rate is significantly influenced by the concentration of F^−^ ions in the electrolyte. In addition, under high-field oxide growth conditions, F^−^ ions can migrate faster than oxygen anions through an oxide layer. This leads to the development of a fluorine-rich layer at the interface between the metal/oxide layer. This layer, rich in fluorine and soluble in aqueous electrolytes, is responsible for transitioning nanopores to hexagonally ordered nanotubes and defining the distance between tube walls (*stage III*). Thus, the water content also affects whether self-organized oxide tubes or pores are formed [33,118,121].

Furthermore, when the metal is transformed into oxide, volume expansion occurs. The degree of this change is defined by the Pilling–Bedworth (PB) ratio. For amorphous titanium and its dioxide, the PB ratio is 2.43, indicating that the volume of formed oxide is more than twice the volume of consumed metal. This volume expansion, along with electrostriction forces, creates localized stress at the interface between the metal/oxide layer, ultimately leading to the shift of the oxide layer from the metallic surface [33,118,121]. Therefore, both the equilibrium between the formation and dissolution of oxide as well as compressive stress are crucial factors in shaping the oxide and fluorine-rich layer into a hemisphere. The theory of self-organization emphasizes individual factors rather than describing the formation mechanism itself, unlike the case of FAD/FAE or ionic and electronic current theory and oxygen bubble mold effect.

All the aforementioned models are being questioned, and a complete theory that explains the entire anodizing process has yet to be established [33,102,116,118,119,121]. The anodizing process of TiO_2_ nanotubes forms the grounds for the formation of TNT membranes, and the process is described for the reader’s sake of understanding. The self-standing titania membranes have a continuous, porous structure. Therefore, the key factor in the nanotube membrane fabrication is the anodizing conditions that determine membrane stability and prevent the structure from cracking [16,122]. The exact anodizing conditions and resulting nanotubes and membrane morphologies are summarized in Table 1, Table 2 and Table 3, in sections describing targeted membrane applications (Section 3.1, Section 3.3 and Section 3.4).

### 2.2. Detachment and Flow-Through TiO_2_ Nanotube Membrane Formation

**Figure 2:**
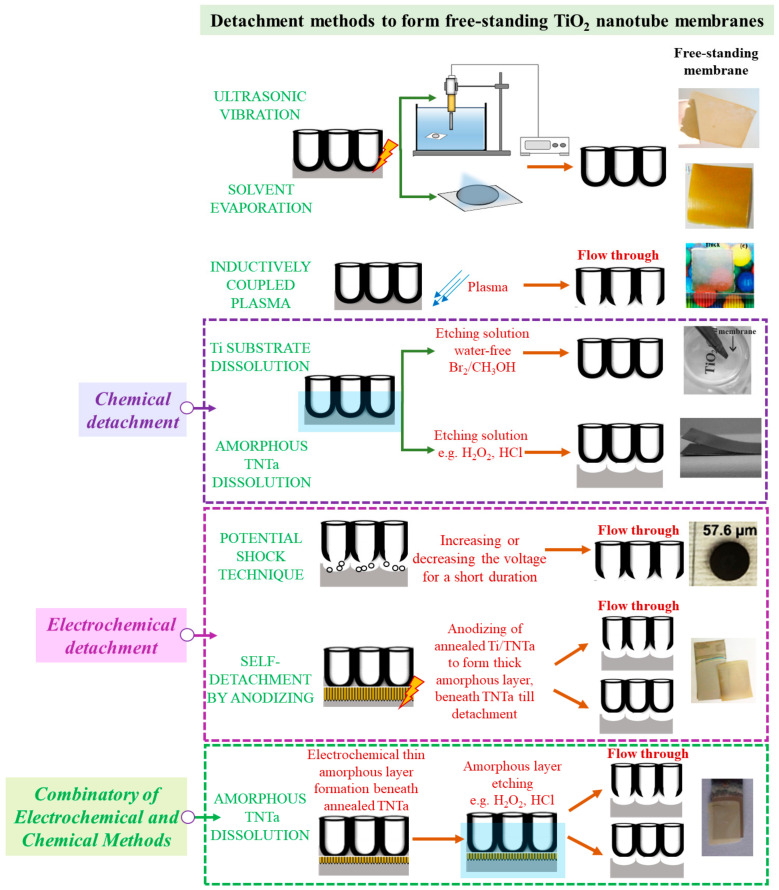
A schematic overview of the different methods to form free-standing TiO_2_ nanotube membranes. Reprinted in part with permission from [60]. Copyright © 2010, American Chemical Society. Reprinted in part with permission from [28]. Copyright © 2014, American Chemical Society. Reprinted in part with permission from [42]. Copyright © 2018, IOP Publishing. Reprinted in part with permission from [97]. Copyright © 2008, John Wiley and Sons. Reprinted in part with permission from [78]. Copyright © 2010, Royal Society of Chemistry. Reprinted in part with permission from [17]. Copyright © 2017, Elsevier. Reprinted in part with permission from [107]. Copyright © 2012, Elsevier. Reprinted in part with permission from [26]. Copyright © 2018, Elsevier.

The process of producing a free-standing TNT membrane involves creating a nanotube array by anodizing and detaching the layer from the metal substrate. Most importantly, the detachment process should preserve membrane integrity. In many applications of titania membranes (e.g., filtration), both side-open nanopores are necessary; therefore, it is very common to find reports dedicated to flow-through membrane formation. The nanotube bottom opening might take place simultaneously with the detachment step or is done separately. Detaching the membrane from the Ti can be achieved through mechanical, chemical, and electrochemical or combinatory methods such as summarized in Figure 2. Additionally, an overview of detachment conditions is placed in Table 1, Table 2 and Table 3, in the following sections dedicated to titania membrane applications (Section 3.1, Section 3.3 and Section 3.4).

A common detachment strategy is to apply an external force to disrupt the adhesion between the TNT membrane and the Ti substrate. This can be achieved using ultrasonic waves, or through the solvent evaporation method to peel off the membrane [20,28,42,60,70,71,72,96,109,123,124,125,126].

In ultrasonication treatment, the detachment process primarily results from cavitation-induced defects and stress at the metal/oxide layer interface. Different solvents can be used for sonication, such as water [60]; methanol [109,123,124]; ethanol [96]; or a mixture of water and ethanol in a ratio of 1:4 (vol/vol) [125].

It was also found that low-surface tension organic solvents such as methanol or ethanol are utilized as rinsing liquids for TNT membrane detachment. After anodizing, the Ti/TNT array is typically washed with an organic bath to remove the electrolyte. However, a small amount of H^+^ and F^−^ can remain between adjacent nanotubes, leading to slow etching of the barrier layer and the formation of defects. Therefore, ethanol, with its low surface tension, can wet the defects. As a result, during slow evaporation, the TiO_2_ nanotube arrays can delaminate due to surface tension [20,42,70,71,72]. In some works, an external mechanical force is applied to the barrier layer in the form of blowing N_2_ gas. Here, methanol was applied as a low-surface tension liquid that can penetrate the barrier layer, while N_2_ gas blowing caused stress and increased the methanol evaporation in pores, leading to cracks and membrane detachment [28,126]. Alternatively, Komba et al. [18] successfully detached the nanotube membrane from the Ti substrate by oven-drying at 75 °C before annealing at 450 °C. The Ti/TNT sample was washed in an ultrasonic ethanol bath before oven- or air-drying. However, the authors have noted that delamination did not occur for the sample dried at room temperature.

Another strategy is applying a rather advanced but very efficient detachment method based on inductively coupled plasma (ICP) etching [42,43]. It is a dry etching process that selectively removes the oxide layer at the bottom of nanotube arrays, leading to free-standing flow-through TNT membrane formation. During the process, high-energy ions from the plasma attack the material and have sufficient energy to break chemical bonds in the barrier layer. As a result, volatile compounds are produced and further pumped out from the ICP system. Compared with other methods, this approach can be used in large areas and eliminate the additional step of opening nanotube-ends.

Among the various chemical detachment methods, the first approach involves the complete chemical dissolution of the Ti substrate by immersing the Ti/TNT in a water-free Br_2_/CH_3_OH solution [37,97]. The authors reported that the resulting membranes had nanotubes with closed bottoms. To open such nanotubes, subsequent exposure to HF vapors was required [37]. However, this procedure is identified as rather time-consuming and involves the use of toxic solutions.

A much simpler chemical separation technique of the TNT array from the Ti substrate is based on the partial chemical-assisted etching of an as-anodized amorphous membrane. It has been reported that the membrane can be detached by short immersion in H_2_O_2_ until it lifts off. Next, the flow-through TiO_2_ nanotube membrane can be produced by placing the membrane over HF vapor [50] or by covering the open side of the nanotubes with a protective layer and immersing it in an oxalic acid solution [78]. Subsequently, the protective layer has been separated from the free-standing flow-through TNT membrane by dipping in an acetone solution [78]. Alternatively, Park and co-workers [69] performed the separation of TNT arrays from metallic Ti substrate by immersing in HCl solution for 1 h. Further nanotube ends openings have not been carried out in this case.

There are frequent reports in the literature that involve electrochemical treatments for TNT membrane detachment. One strategy uses the potential shock method that involves increasing or decreasing the voltage for a short duration at the end of the anodizing process [12,13,85,92]. The change causes electrolyte acidification and gas evolution at the metal/oxide layer interface, which ultimately results in the detachment and opening of tube ends. This method takes advantage of stress formation between two layers, making it a convenient and effective technique that eliminates the need for additional etching to open tube bottoms. Cha et al. [12,13] used this method after anodizing in 0.15 M NH_4_F/ethylene glycol electrolyte for an hour at 60 V, subsequently rising the voltage to 120 V for 3 min. In a different research, Liao and co-workers [85] anodized a Ti substrate in a mixture of 0.3 wt% NH_4_F and 2 vol% deionized water in ethylene glycol at 60 V for 3 h to form the TNT and increased the voltage to 150 V for 5 min to fabricate a free-standing flow-through TNT membrane. Alternatively, the potential shock method can be applied not at the end of the original anodizing but as the following step. It was shown that after finishing TNT formation and samples rinsing, the membrane detachment was achieved by applying a high voltage of 180 V for 3–5 min [92]. To complete the removal of the barrier layer that blocks certain pores the authors performed etching in buffer oxide solution.

Another popular electrochemical membrane detachment strategy is based on an extra anodizing step after accomplishing the nanotube formation. Most typically, first, the original nanotubes are annealed to stabilize the layer, and next, the titanium substrate undergoes anodizing till the top layer with nanotubes is detached [19,22,23,27,32,51,68,107,127]. The conditions applied for the subsequent anodizing steps might be the same or different (electrolyte, potential, time and/or temperature). Some works have shown that by controlling the annealing temperature, it is possible to directly create free-standing TNT membranes with both open tube ends [14,19,127]. For example, Lin et al. [127] conducted detachment of that TNT obtained in a two-step anodizing procedure by so-called third anodizing. In this work, unannealed and annealed TiO_2_ layers on Ti substrate (between 0 and 700 °C for 3 h) were anodized under the same potentiostatic conditions (60 V) at a higher electrolyte temperature of 30–50 °C for 0.5–1 h. For the oxide layer annealed at 200 °C for 3 h (to achieve partial crystallization), the third anodizing step led to self-detachment with both nanotube’s ends being open, but post-treatment annealing was necessary to obtain a fully crystallized membrane. When the nanotubes annealed at 400 °C were used for the third anodizing step, the TNT membrane was formed with closed nanotube bottoms. The authors also noted that the TiO_2_ nanotube array could not be detached from the Ti substrate when subjected to a lower temperature thermal treatment at 100 °C. Moreover, it has been observed that the detaching time can be reduced by increasing the bath temperature during the third anodizing process as a result of decreased viscosity and enhanced current density [127].

Additionally, the composition of anodizing electrolytes used in self-detachment plays an important role in affecting the etching rates, surface reactions and dynamics, and stress distribution at the interface. For instance, Peighambardoust and co-workers [27] found that the detachment occurred faster (after ~20 min) when fresh ethylene glycol electrolyte was used instead of an aged (reused) one.

One of the most popular detachment methods combines electrochemical amorphous layer formation and chemical etching steps. Analogously to the electrochemical self-detachment method, first, the crystalline titania nanotubes on the Ti substrate undergo additional anodizing to form a thin amorphous layer beneath the nanotubes. In the following step, the amorphous layer is chemically etched due to less chemical resistivity, while crystalline nanotubes remain unaffected. To selectively dissolve the amorphous lower layer, various solutions, such as H_2_O_2_ [21,24,26,45,49,52,53,54,55,56,62,74,83,84,99,110,128] or HF [12,15,25,73], have been used.

Whether the formed nanotubes have one or both open ends depends on the annealing temperature, which was also emphasized with the self-detachment method [14,17,19,35,127]. Thus, when the annealing temperature was up to 300 °C, partial crystallization occurred, and further anodizing and etching in H_2_O_2_ resulted in the flow-through, free-standing TNT membrane formation [17]. In other works, pre-crystalized annealing followed by short chemical etching in H_2_O_2_ enabled the lifting of a free-standing TNT membrane but with closed bottom tube ends. To obtain a flow-through TNT membrane, additional annealing at 450 °C for 1 h and submerging in an oxalic acid solution was necessary [35,36]. According to Rezaei and co-workers [26], the voltage applied during the re-anodizing of a pre-crystalized TNT array on the Ti substrate strongly affected the flow-through pores formation. By performing the third anodizing at 100 V, followed by chemical etching in H_2_O_2_, free-standing TiO_2_ nanotube membranes with both ends open were prepared. However, when the anodizing was conducted under 80 V, the TNT membrane with closed bottom ends was produced [26]. Some works also reported the preparation of the free-standing membrane through the chemical dissolution of the amorphous layer formed by anodizing, but to open the bottom ends of the TNT membrane, an extra procedure such as ion milling with Ar^+^ bombardment was involved [38,39,40,41].

## 3. Applications of TiO_2_ Nanotube Membranes

### 3.1. Photocatalytic and Electrocatalytic Hydrogen Generation

TiO_2_ is the benchmark *n-type* semiconductor material with suitable conduction (CB) and valence band (VB) edge positions that are prerequisites for the solar-driven photolysis of water and/or organic compounds. The application of TiO_2_ towards photocatalytic and/or photoelectrochemical (PEC) hydrogen generation from water splitting has been continuously revised, starting with the pioneering work of Fujishima and Honda in 1972 [129]. In the study, a small external bias and light illumination was applied to generate hydrogen on the platinum cathode, while titania photoanode produced oxygen.

Giant progress has been achieved through the use of TiO_2_ in the forms of various nanostructures, such as nanopowders or nanotubes/nanoporous layers, as a quantum confinement effect and a great surface-to-volume ratio enormously improve their functionality and unique properties [2,130]. Anodic TNT layers show a bunch of beneficial features for PC/PEC applications; for example, they have a huge total surface area available for a reaction that is incomparably higher than in compact materials. The major drawbacks limiting the process’s performance are the large band gap of the pure TiO_2_ (only UV light absorption), the recombination of photogenerated charges, and the slow kinetics of reactions occurring on TiO_2_/aqueous environment interfaces.

When used as a photocatalyst for water splitting, under open-circuit conditions, TiO_2_ structures typically require a co-catalyst deposition for reasonable H_2_ production yield due to oxidation and reduction reactions occurring at the same material surface [131,132]. The presence of co-catalysts is necessary to enable spatial separation of the two processes and frequently catalyze the reaction (e.g., noble metals are hydrogen reduction catalysts). Combining titania with secondary materials is the most common strategy of materials modifications, although more focus is also on nanostructured materials architecture. In the literature, considerable attention has also been given to forming and modifying one-dimensional nanostructures, such as free-standing TiO_2_ nanotube membranes. That opens a new perspective for further increasing the surface area available for the reaction and for materials site-selective design to enhance light-induced photocatalytic reactions (e.g., water splitting) [3].

The summary of recently published results on free-standing and flow-through TiO_2_ nanotube membranes application for hydrogen production is collated in Table 1. Table 1 presents details on titania membranes fabrication, their modification, experimental conditions, and reported hydrogen production efficiency.

**Table 1:** Summary of preparation and application of free-standing/flow-through TiO_2_ nanotube membranes applied for hydrogen generation.

Anodizing Parameters	TNT Layer Detachment	TNT Layer Morphology			Photocatalytic Hydrogen Production					Ref.
		Thickness/Lenght (μm)	Tube Diameter (nm)	Wall Thickness (nm)	Catalyst Preparation	Electrolyte	Measurement Conditions		Results	
							Photocatalyst	Light Source	H_2_ Production Rate	
0.15 wt% NH_4_F, 3 vol% H_2_O in EG 1st: 60 V for 1 h	- Annealing at 350 °C for 1 h; - Anodizing for 60 V for 1 h; - Amorphous layer chemical dissolution in 0.07 M HF	15	110	-	- Attached to FTO glass with doctor-bladed TiO_2_ NPs paste - Deposition of 5 nm Pt layer by plasma sputtering - Annealed at 500 °C for 1 h	20 vol% methanol aqueous solution	TNTs decorated with Pt NPs	HeCd laser (λ = 325 nm, 60 mW cm^−2^) or CW-laser (λ = 266 nm, 11.9 mW cm^−2^)	325 nm laser: grass top TiNTs ~126 μmol h^−1^; 266 nm laser: grass top TiNTs ~30 μmol h^−1^;	[12]
	- Annealing at 350 °C for 1 h - Anodizing for 60 V for 1 h - Amorphous layer chemical dissolution in 0.07 M HF				- Attached to FTO glass with doctor-bladed TiO_2_ NPs paste - Annealed at 350 °C for 30 min and ultrasonicated for 20 min - Deposition of 5 nm Pt layer by plasma sputtering - Annealed at 500 °C for 1 h				325 nm laser: open top TiNTs ~144 μmol h^−1^; 266 nm laser: open top TiNTs ~38 μmol h^−1^;	
	- Chemical dissolution by immersing in ethanol				- Flipped over and/or attached to FTO glass with doctor-bladed TiO_2_ NPs paste - Deposition of 5 nm Pt layer by plasma sputtering - Annealed at 500 °C for 1 h				325 nm laser: bottom closed TiNTs ~42 μmol h^−1^; 266 nm laser: bottom closed TiNTs ~16 μmol h^−1^;	
0.15 wt% NH_4_F, 3 vol% H_2_O in EG 1st: 60 V for 1 h	- Potential shock technique (120 V for 3 min) and soaking in ethanol								325 nm laser: bottom open TiNTs ~70 μmol h^−1^; 266 nm laser: bottom open TiNTs ~23 μmol h^−1^;	
0.15 wt% NH_4_F, 3 vol% H_2_O in EG 1st: 60 V for 1 h	- Potential shock technique (120 V for 3 min) and soaking for 20 min in ethanol	15	outer diameter ~100–120;inner diameter ~30–40	~60–70	- Transferred on a ceramic block, coated with an FTO glass slide with different configuration; dried overnight; - Deposition of Pt layer by plasma sputtering - Flipped over and/or attached to FTO glass with doctor-bladed TiO_2_ NPs paste (2.5 µm) - Annealed 500 °C for 1 h	20 vol% methanol aqueous solution	TNTs decorated with or without Pt NPs	HeCd laser (λ = 325 nm) 60 mW cm^−2^; active area 0.78 cm^2^	- TNTs membrane without Pt: front-side illumination ~3.4 µL h^−1^ cm^−2^ and: back-side illumination ~1.5 µL h^−1^ cm^−2^; TU 5,5 F ~241 µL h^−1^ cm^−2^ TU 5,5 B ~93 µL h^−1^ cm^−2^ TU 0,10 F ~74 µL h^−1^ cm^−2^ TU 0,10 B ~71 µL h^−1^ cm^−2^ TU 5,0 F ~148 µL h^−1^ cm^−2^ TU 5,0 B ~57 µL h^−1^ cm^−2^ TU 0,5 F ~74 µL h^−1^ cm^−2^ TU 0,5 B ~66 µL h^−1^ cm^−2^ TU 10,0 F ~174 µL h^−1^ cm^−2^ TU 10,0 B ~60 µL h^−1^ cm^−2^ TD 5,5 F ~163 µL h^−1^ cm^−2^ TD 5,5 B ~178 µL h^−1^ cm^−2^ TD 0,10 F ~121 µL h^−1^ cm^−2^ TD 0,10 B ~46 µL h^−1^ cm^−2^ TD 5,0 F ~82 µL h^−1^ cm^−2^ TD 5,0 B ~133 µL h^−1^ cm^−2^ TD 0,5 F ~98 µL h^−1^ cm^−2^ TD 0,5 B ~39 µL h^−1^ cm^−2^ TD 10,0 F ~81 µL h^−1^ cm^−2^ TD 10,0 B ~142 µL h^−1^ cm^−2^ *TU/D X,Y F/B: TU and TD– top up and top down tube configuration onto FTO, respectively; X,Y—nominal thickness (in nm) of the sputtered Pt film at top and bottom of tubes, respectively F and B stand for front- and back-side illumination, respectively	[13]
0.15 M NH_4_F, 3 vol% H_2_O in EG 1st anodization step Removed by ultrasonication in water 2nd anodization step	- Annealing at 250 °C for 2 h; - 3rd anodizing; - Amorphous layer chemical dissolution/etching in 30 wt.% H_2_O_2_ for 3 h at room temperature	1.5–60			- Soaked in methanol - Flipped over and attached to FTO glass with doctor-bladed TiO_2_ NPs paste (2.5 µm; -TiO_2_ NPs layer was annealed at 250 °C for 20 min) - Deposition of 5 nm Pt layer by Ar-plasma sputtering - Annealed at 450 °C for 1 h in air with a rate of 30 °C min^−1^	20 vol% methanol aqueous solution	TNTs decorated with or without Pt NPs	HeCd laser (λ = 325 nm) 60 mW cm^−2^; active area 0.785 cm^2^		[17]
glycerol and distillate H_2_O in a ratio of 4:1, including 0.5 wt% of NH_4_F 1st: 50 V for 10 h	- Mechanical detachment/peeling-off	5	100–200	-	- Annealed at 500 °C in air for 2 h - Coated with a Pd film via electroless plating - Annealed at 500 °C for 5 h under N_2_ atmosphere	Mixture of pure water and methanol in a ratio of 1:1	3 µm thick TNT/Pd membrane (1 µm thick TNTa coated with 2 µm thick Pd film)	Xe lamp (λ = 300–400 nm) 30 mW cm^−2^, 1.5 cm diameter of active area	0.21 µmol^−1^ h^−1^ cm^−2^	[44]
glycerol and distillate H_2_O in a ratio of 9:1, including 0.5 wt% of NH_4_F 1st: 50 V for 10 h		5	100	-	- Pd sputtering at 400 °C for 4 h		TNT coated with 10 µm Pd film		-	
0.3 wt% NH_4_F, 2 vol% H_2_O in EG 1st: 60 V for 10 h	- Mechanical detachment/solvent evaporation; N_2_ blowing combined with methanol wetting	110	90	-	- Annealed at 500 °C for 30 min in air - Transformation into 3D and 1D/3D hybrid composition by peroxide etching and hydrothermal treatment in NaOH	Water and methanol mixture with a ratio of 9:1 (vol/vol)	40 mg (catalyst to solvent ratio of 1 g/L)	300 W Xe lamp, UV irradiation, 1.8 mW cm^−2^	Annealed TiO_2_ nanotube membrane, HHC and 3D-HTiO_2_ decorated with 1% of Pt was 385 μmol h^−1^ g^−1^, 661 μmol h^−1^ g^−1^ and 1310 μmol h^−1^ g^−1^, respectively	[28]
0.3 wt% NH_4_F, 2 wt% H_2_O in EG, RT 1st: 60 V for 1 h Removed by ultrasonication in 0.1 M HCl 2nd: 60 V for 4 h	- Solvent evaporation/drying in the oven at 75 °C	38	Inner diameter 95; outer diameter 120	24 ± 1.1	- Annealed at 450 °C in the air muffle furnace for 2 h (rate 10 °C min^−1^) or exposure to hot water steam for about 30 min - 60 mg of TNTs was dispersed in ethanol and mixed with 32 mg of MoS_2_, sonicated for 10 min in ice water, dried in 95 °C - Dried powder annealed at 480 °C for 2 h (MoS_2_/TiO_2_-NTs)	0.5 M H_2_SO_4_	5 µL of the catalyst ink (350 µL of H_2_O/EtOH (5:3 vol/vol) with 5 wt% Nafion 0.4 ratio) immobilized onto glassy carbon electrode; geometric surface area 0.2475 cm^2^ loading of the catalyst 0.450 mg cm^−2^ on carbon fiber paper	-	-	[18]

Wierzbicka et al. [45] proposed a very promising approach to preparing the PEC system for water splitting. The composite core-shell photoanode is based on a free-standing TiO_2_ membrane (detached from Ti by chemical etching) serving as the light absorber, filled with electrodeposited Au nanowires acting as the electron collector. The photon-to-current efficiency of the resulting photoanode reaches 35%, surpassing that of a reference material produced under identical anodizing conditions by fourfold. The reported material exhibited exceptional stability, evidenced by no loss in photocurrent over 100 on-off cycles of light illumination, continuous exposure to light for 12 h, and one-month storage in air. This significant enhancement in performance was attributed to the distinctive architecture of the photoanode, which facilitates electron separation through the presence of a Schottky-type contact and enables rapid electron transport via the Au nanowires.

Cha, Schmuki, and their colleagues [12] analyzed the relationship between morphology and photocatalytic properties of self-organized TiO_2_ nanotube membranes immobilized on a fluorine-doped tin oxide (FTO) glass and decorated by co-catalyst platinum towards hydrogen generation from photocatalytic water splitting. The authors differentiate between two ways of preparing TiO_2_ nanotubes using an anodic oxidation process. The two-step anodizing was conducted in an F-based ethylene glycol electrolyte using a constant voltage of 60 V for 1 h with annealing at 350 °C for 1 h in between the steps to minimize layer damage during detachment. By dipping the samples in 0.07 M fluoric acid, the researchers separated the TiO_2_ nanotube layer with collapsed etched tube residues (the first type samples). Furthermore, annealing at 350 °C for 30 min, followed by ultrasonication, has been applied to open the top layer of nanotubes (the second type sample). The third type sample, also obtained during the two-step anodizing process, was detached in ethanol and characterized by closed bottom tubes that were exposed to the electrolyte. The fourth TiO_2_ nanotube membrane sample was obtained by increasing voltage at the end of the first anodizing step (120 V for 2 min), resulting in open bottom ends. Based on SEM images, the TNT layers were found to have approximately 110 nm in diameter and a length of about 15 µm. The work has confirmed that the unique open tubular structure of TiO_2_ allows for better Pt dispersion within the structure, enabling sufficient light penetration. As a result, the hydrogen generation rate is 3.5 times higher than the value obtained for bottom-closed TNTs directed towards the electrolyte. In addition, when comparing the results obtained from an open tubular structure of TNTs that were differentiated based on Pt loading, it was found that the highest hydrogen yield (~144 μmol h^−1^; λ = 365 nm) was achieved with Pt nanoparticles (NPs) formed by dewetting a 5 nm Pt layer. This clearly indicates that the photocatalytic activity of TNTs is affected by the nanotube configuration [12].

**Figure 3:**
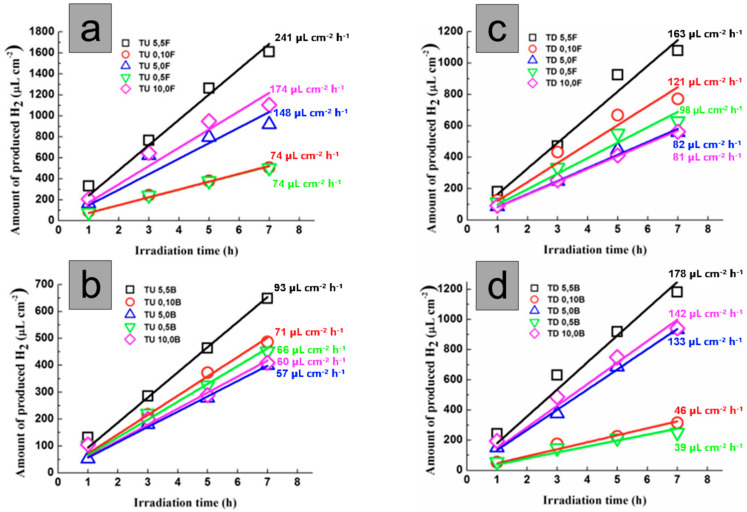
Photocatalytic results in terms of amount of generated H_2_ vs. irradiation time of Pt-decorated TNT membranes in a tube top-up ((**a**,**b**)) and top down ((**c**,**d**)) under front- ((**a**,**c**)) and back-side illumination. Reprinted in part with permission from [13]. Copyright © 2016, John Wiley and Sons.

Another research performed by Cha et al. [13] showed that the noble metal’s particle size, location, and decoration density of TNT’s membranes are also important factors influencing photocatalytic water splitting. The authors have shown the rate of hydrogen generation increased up to 70 times when both sides of the TiO_2_ nanotube membrane were decorated with Pt (TNTs membrane ~3.4 µL h^−1^ cm^−2^ and TNTs membrane decorated on both sides with 5 nm of Pt ~241 µL h^−1^ cm^−2^). The central part of the membrane functions as a light-harvesting zone, while NPs co-catalysts act as electron trapping centers (formation of Schottky junctions), improving the charge carriers’ separation. However, it was pointed out that excessive deposition of nanoparticles on TiO_2_ nanotubes can hinder light penetration, causing a decline in activity. Proper photocatalyst configuration is crucial for preventing Pt shading effects and reaching optimal catalytic performance. Moreover, the positive impact of Pt-decorated nanograss on the photocatalytic activity toward H_2_ generation has been highlighted. This spatial structure acts as a three-dimensional TiO_2_ network with voids and empty spaces, allowing for the deeper deposition of noble metal into the structure and homogenous coats of the uppermost part of the TNT membrane. It has also been emphasized that the path of light irradiation (front- and back-side irradiation) significantly impacts the photocatalytic performance of the TNT membranes (Figure 3). When Pt is deposited at the opposite end of the membrane concerning the illumination pathway, the probability of charge carrier recombination increases; thus, photocatalytic efficiency decreases. This is due to the photogenerated charge carriers’ long diffusion pathway to reach the membrane’s co-catalyst-rich side and participate in the photocatalytic reaction.

**Figure 4:**
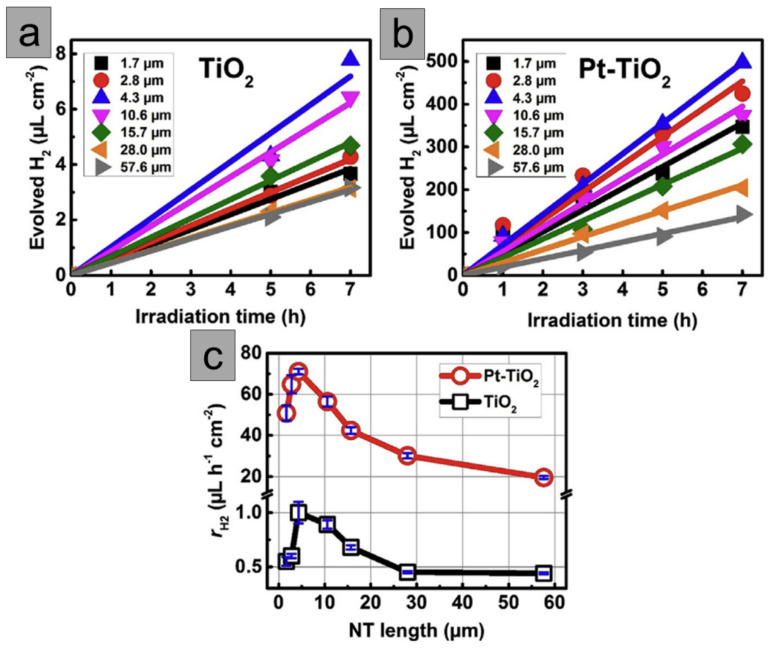
Amount of photocatalytically evolved H_2_ measured over irradiation time for (**a**) pristine and (**b**) Pt-decorated TiO_2_ nanotube membranes of different thicknesses; (**c**) photocatalytic results expressed in terms of H_2_ evolution rate (rH_2_) for pristine and Pt-decorated TiO_2_ nanotube membranes plotted as a function of the membrane thickness. Reprinted in part with permission from [17]. Copyright © 2017, Elsevier.

The impact of the thickness of free-standing TiO_2_ nanotube membranes on photocatalytic hydrogen generation was investigated [17]. It was observed that the diffusion distance that electrons and holes have to travel to reach the interface must be optimized to find the highest charge carrier separation. The researchers produced and analyzed TNT membranes in the form of anatase, with thickness in a range of 1.5 to 60 µm. The nanotubes were further immobilized onto FTO (with the nanotube top side facing the conducting glass) and decorated with Pt NPs. In order to achieve a maximized photocatalytic efficiency, it has been observed that a nanotube length of 5–10 µm is optimal due to its efficient electron transfer kinetics (diffusion over a short distance). A notable decline in the hydrogen evolution efficiency has been observed for membranes thicker than approximately 30 µm due to the increase in recombination rate. If the thickness of TNT layers was greater than 20 µm, the nanograss structure formation was a result of etching that takes place during prolonged exposure to F-containing electrolyte. The authors highlighted that the outer nanotube diameter exhibits a negligible increase with increased anodizing time. As per previous studies, it has been observed that the presence of Pt NPs led to a significant increase in photocatalytic activity. In the case of bare TNT membrane, the H_2_ evolution rate showed only a slight difference with the increase in the membrane thickness (between 0.45 and 1 µL H_2_ h^−1^ cm^−2^), while Pt-TNT membranes were influenced much stronger (30 and 70 µL H_2_ h^−1^ cm^−2^) (Figure 4). These details have been explained by Schottky junction formation and directional diffusion gradient within TNT walls in the presence of Pt NPs.

An alternative approach for producing H_2_ through photocatalysis has been proposed by Hattori and Noda [44]. The authors used TNT membranes coated with a palladium (Pd) film to decompose methanol under UV light irradiation and to separate hydrogen gas from other byproducts such as formaldehyde, formic acid, carbon monoxide and/or carbon dioxide. It has been determined that thinner and defect-free layers of Pd (2 µm thick) over TNTs can be produced using the electroless plating method, compared to the physical vapor deposition method. Therefore, the researchers estimated that the amount of hydrogen produced from the methanol/water mixture over the TNTs/electroless-plated Pd membrane per unit area of the membrane was estimated to be 0.21 µmol^−1^ h^−1^ cm^−2^ during the initial 2 h of the UV illumination.

According to Kang and Haider’s assessment [28], modifying the morphology of TiO_2_ nanotubes’ and doping with Pt NPs had a significant effect on the photocatalytic activity towards hydrogen generation from water under UV irradiation in the presence of sacrificial agent, methanol. The TiO_2_ membrane was prepared by an electrochemical anodizing process in fluoride electrolyte and detached from the Ti substrate by methanol wetting. After being formed as amorphous, the TiO_2_ nanotube membrane was crystallized to an anatase structure (Cryst-TiO_2_-Memb) through annealing at 500 °C for 5 h. Furthermore, the researchers proposed transforming an initial TiO_2_ nanotube membrane into 3D hierarchical TNTs (3D-HTiO_2_) and 1D/3D hybrid composite structures (HHC) to enhance the material’s active surface area and overall activity. The formation of 3D hierarchical TNTs involved etching an amorphous TiO_2_ membrane in H_2_O_2_ solution followed by hydrothermal transformation in NaOH solution. Then, acid washing replaced sodium cations with hydrogen ones, followed by thermal annealing in the air to form a crystalline phase. The HHC sample was formed by hydrothermal treatment of Cryst-TiO_2_-Memb in NaOH, which was then subjected to post-annealing. The catalysts mentioned above, all decorated with Pt nanoparticles, were utilized for photocatalytic hydrogen generation from water splitting. The average H_2_ production observed by annealed TiO_2_ nanotube membrane (Cryst-TiO_2_-Memb), HHC and 3D-HTiO_2_ was 385 μmol h^−1^ g^−1^, 661 μmol h^−1^ g^−1^ and 1310 μmol h^−1^ g^−1^, respectively. The authors claim that increasing the specific surface area of hierarchical TiO_2_ can enhance H_2_ production while reducing photogenerated charge carrier recombination. However, it is noteworthy that the improvement of active surface area results in a better dispersion of Pt nanoparticles and increased Pt loading.

Despite the beneficial effects, such as improved photo-induced charge carrier separation and transport resulting from noble metal co-catalyst deposition on TNT membranes, there is a constant need to lower the cost of hydrogen production. It might be obtained by intensifying research towards equally efficient alternatives for noble metals and/or reducing the amount of noble metal used, for instance by single-atom formation.

In research conducted by Sun and colleagues [18], it was observed that covering the surface of a free-standing TiO_2_ nanotube membrane with a layer of MoS_2_ resulted in enhanced hydrogen generation from electrochemical water splitting. The resulting system, called MoS_2_-supported free-standing (MoS_2_/TiO_2_-NTs), demonstrated improved hydrogen evolution reaction (HER) performance in acidic media with a low overpotential of 170 mV at which 10 mA cm^−2^ current density is achieved. Additionally, the composite system showed a smaller Tafel slope (70 mV decade^−1^) as compared to the individual components, with MoS_2_ and TiO_2_-NTs having Tafel slopes of 120 and 200 mV decade^−1^, respectively. The MoS_2_/TiO_2_-NTs arrangement also showed long-term stability (5000 cycles) between −0.4 and 0.2 V vs. RHE potential range. The improved HER performance of the composite (MoS_2_/TiO_2_-NTs) has been attributed to its large surface area and the synergetic effect arising from the chemical and electronic interactions between the TiO_2_-NTs and MoS_2_. However, in electrochemical water splitting, an external voltage is applied to the electrodes as a driving force, which increases the cost of production. 

Diverse advanced and well-known characterization methods have provided comprehensive information about materials properties. X-ray diffraction (XRD) remains a fundamental tool in investigating phase composition and crystal structure [12,13,17,18,28,44,45]. Scanning electron microscopy (SEM) analysis has been performed to characterize the morphology and to confirm the expected dimensions, vertical uniformity, and surface roughness of the nanotube membranes [12,13,17,18,28,44,45]. Transmission electron microscopy (TEM) makes it possible to obtain information about interplanar lattice spacing and morphology, as well as the crystal structure [13,18,28,45]. In some cases, researchers have provided information about material compositions by combining SEM with energy dispersive spectroscopy (EDX) [13,28,45]; X-ray photoelectron spectroscopy (XPS) [12,13,17,18,28,44,45]; or inductively coupled plasma (ICP) spectrometry when modified with Pt [13]. Wierzbicka et al. [45] applied the selected area electron diffraction (SAED) method for local phase identification, the results of which were supplemented by XRD material analysis. In addition, researchers used electrochemical impedance spectroscopy (EIS), Mott–Schottky analysis, and ultraviolet photoelectron spectroscopy (UPS) to characterize the electronic properties of TiO_2_ nanotube membranes [45]. Optical properties were studied by using UV–Vis diffusive reflectance spectroscopy (DRS) [12,28] and/or incident photon-to-electron conversion efficiency (IPCE) [45]. For (photo)electrochemical characterization of the materials based on TNT membranes the voltammetry method has been utilized [12,13,17,18,28,44,45]. The Brunauer–Emmett–Teller (BET) theory, based on the assumption that gas as molecules form monolayer adsorption, was used to determine the specific surface area of TNT membranes [18,28]. In addition, the amount of H_2_ photocatalytically generated under irradiation has been determined by gas chromatography (GC) equipped with thermal conductivity detector (TCD) detector with argon as a carrier gas [12,13,17,44].

### 3.2. Fuel Cells

Another type of TNT membrane application has been found in fuel cells. They are proposed to replace carbon-based support materials in polymer electrolyte membrane fuel cells (PEMFCs). The main component of PEMFCs is the membrane electrode assembly (MEA), which consists of a polymeric electrolyte membrane (PEM) and two electrodes—an anode and a cathode—arranged in a sandwich-like structure. Conventionally, carbon material has been utilized as a support for catalyst nanoparticles, such as Pt or Pt alloys in PEMFC electrodes arrangement. However, the carbon structures are characterized by weak bonding with metal and tend to corrode during operation, which leads to PEMFC electrode degradation. In contrast, titanium dioxide is characterized by high corrosion resistance and good interaction with metal catalysts. Thus, the utilization of TNT membranes as an alternative to conventional carbon-based catalyst support has been proposed. Nonetheless, the relatively poor electronic conductivity of TiO_2_ nanotubes needs to be addressed. Zhang et al. [46] proposed an ultrathin catalyst layer derived from a free-standing TNTs membrane for PEMFCs application to meet the requirements mentioned above. To improve the electrical conductivity, TiO_2_ nanotubes were decorated with carbon using the plasma-enhanced chemical vapor deposition (PECVD) technique. The electrical conductivity of C-TNTs was measured to be 300 S cm^−1^, while that of TNTs was found to be ~1.4 · 10^−4^ S cm^−1^. The carbon-coated TiO_2_ electrode, which was sputtered with Pt, was hot-press onto Nafion 211 membrane to separate from the metal substrate and further etched in the diluted HF. When used as a cathode, C-TNTs-Pt with ultralow metal loading of 24.8 µg_Pt_ cm^−2^ and 50.1 µg_Pt_ cm^−2^ achieved a maximum power density of 206 and 305 mW cm^−2^, respectively, in a single fuel cell (cell temperature 65 °C; fully humidified H_2_/O_2_ with a backpressure of 0.05 MPa). In comparison, a commercial Pt/C gas diffusion electrode (GDE) with a Pt loading of 0.4 mg_Pt_·cm^−2^ exhibited a power density of 983.1 mW·cm^−2^ under the same testing conditions [57]. In addition, when measuring the oxygen reduction reaction (ORR) in 0.5 M sulfuric acid solution, it was indicated that C-TNTs-Pt with Pt loading of 24.8 µg_Pt_ cm^−2^ exhibited significantly higher activity compared to those without carbon coating. The electrochemical specific surface area (ECSA) of C-TNTs-Pt was reported as 42.8 m^2^ g^−1^_Pt_, and the mass activity at 0.85 V was found to be ~0.44 A mg_Pt_^−1^. Manikandan et al. [124] demonstrated the potential use of TNT membrane decorated with Pt NPs by chemical impregnation method in PEMFCs for oxygen and methanol oxidation reaction (MOR). The electrochemical specific surface area of Pt/TNT was calculated to be 75 m^2^ g^−1^_Pt_. The high durability of Pt/TNT has been confirmed by a low reduction of only 12% in ECSA after 1000 cycles, compared to a 76% and 40% reduction for Pt/C and Pt/TiO_2_ NPs, respectively. Moreover, Pt/TNT was characterized by much higher mass activity of 0.86 A mg^−1^_Pt_, which was almost 17 times higher than that of Pt/C and three times higher than that of Pt/TiO_2_ NPs. Researchers attributed this to the high surface area, porous nanotube structure, and strong interaction between metal and TiO_2_ nanotubes. An efficient charge transfer was attributed to the presence of oxygen defects in TNT nanotubes. In addition, the strong interaction between metal-TNTs substrate followed by enhanced charge transfer resulted in improved Pt/TNT performance in MOR compared to Pt/TiO_2_ NPs and Pt/C.

As expected, the structure, dimensions, and morphology of synthesized samples have been characterized by SEM and TEM imaging and the crystallographic structure and chemical composition analysis has been performed by XRD, XPS, SEM-EDX, ICP spectroscopy, and Raman spectroscopy [46,57,124]. Manikandan et al. [124] utilized photoluminescence (PL) spectroscopy to analyze the surface and interface of the bare TiO_2_ and, after modifying with Pt. More detailed characterization of fuel cell catalyst activity has been provided by current-voltage measurements, cyclic voltammetry (CV), and EIS measurements [46,57,124]. In addition, CV, EIS, as well as BET methods, have been utilized for surface area determination being a crucial fuel cell catalyst parameter.

### 3.3. Li-S Batteries

Lithium-sulfur (Li-S) batteries are considered to be efficient energy storage systems due to their high energy density of 2500 Wh kg^−1^ and a specific capacity of 1675 mAh g^−1^ [133]. However, the utilization of sulfur as the cathode material in Li-S cells is limited because of the sulfur’s insulating nature and the dissolution of polysulfides. These issues have a negative impact on the battery’s capacity and cycling performance. To address this, Dasarathan and co-workers [83] proposed the use of a TiO_2_ nanotubular hybrid membrane as a polysulfide scavenger to effectively bond S-species and improve battery performance. The free-standing and flow-through TNT membrane was fabricated by a two-step anodizing, followed by chemical etching of the amorphous underlayer. The highly porous nanograss structure on the tops of the nanotubes was formed by chemical engravings during anodizing while, double-walled TiO_2_ nanotubes were formed at the bottom membrane part (after annealing at 450 °C). Before application in an Li-S battery, a thin, insulating polymer layer was spin-coated on the top of the TiO_2_ nanoporous grassy structure for membrane protection. The TiO_2_ nanograssy tubular membrane (with a nanotube thickness of ~25 µm and diameter of 140–180 nm) exhibited an overall capacitance of 852.4 mA h g^−1^ after 5 cycles at 0.39 mA cm^−2^. Furthermore, the cell containing the TiO_2_ nanograssy tubular membrane demonstrated notable cycling stability over 100 cycles, high coulombic efficiency of >97%, and a high discharge capacity of 618 mA h g^−1^ compared to the Li-S battery using a commercial polyethylene separator (performance dropping to zero after 40 cycles). Morphological characterization of the free-standing nanograssy tubular hybrid membrane has been performed using SEM and TEM analysis. The XRD technique provided information about the composition and crystal structure of the TiO_2_ nanotube layer. Cyclic voltammetry, galvanostatic charge-discharge, and electrochemical impedance spectroscopy techniques were utilized to investigate the behavior of TiO_2_ nanograssy tubular membrane in an Li-S cell [83]. However, a systematic investigation by in situ (operand) techniques seems necessary for a proper understanding of the reaction mechanism in Li-S cells, even though it often requires high-cost facilitates.

Indeed, the results show that the TiO_2_ nanotube membranes serve as an efficient scavenger for polysulfide, reducing the active material degradation and allowing at the same time Li-ions to diffuse into the cathode, which leads to improved capacity and cycling behavior. This seems to suggest that the TiO_2_ nanotube membranes would be an effective means of introducing low-cost, environmentally friendly, and abundant Li-S batteries in a large-scale application.

### 3.4. Dye-Sensitized Solar Cells

Dye-sensitized solar cells (DSSCs) have gained scientific interest as cost-effective devices that convert solar energy into electricity. Typical DSSC is composed of three elements: (i) photoanode with dye sensitizer-covered semiconductor coating a conductive transparent substrate (e.g., fluorine-doped tin oxide coated glass); (ii) a counter electrode (Pt coated); and (iii) an electrolyte (containing I^−^/I^3−^ redox couple). Under solar illumination, the sensitizer—dye with a broad absorption band present on the surface of a wide-band semiconductor—harvests the light [27]. The photo-induced electrons are injected from the (lowest unoccupied molecular orbital) LUMO of the dye into the semiconductor’s conduction band and transported to the charge collector [134]. Subsequently, the dye is reduced by electrons from the electrolyte, which is also further reduced, and the counter electrode closes the circuit.

The overall performance of the DSSCs is typically described by multiple parameters, i.e., dye loading, short-circuit current density (*J_SC_*), open-circuit voltage (*V_OC_*), fill factor (*FF*), and overall energy conversion efficiency (*η*). The latter two can be calculated using Equations (1) and (2) [27]:

(1) $$FF=\frac{P_{max}}{J_{SC}V_{OC}}=\frac{I_{max}V_{max}}{J_{SC}V_{OC}}$$

(2) $$\eta =\frac{J_{SC}V_{OC}FF}{P_{in}}\;*\;100$$

where *P_in_* is the light power per unit area, *I_max_* and *V_max_* are current and voltage at the maximum power in *J-V* curves measured during DSSCs performance under illumination. The *P_in_* is usually 100 mW cm^−2^ when a standard AM 1.5 solar simulator is used. The light-harvesting efficiency depends on the number of dye molecules attached to the semiconductor surface; therefore, semiconductors with a highly developed surface area are desirable in DSSCs construction.

The first proposal of using TiO_2_ nanoparticles in dye-sensitized solar cells to convert solar energy into electricity was reported in 1991, achieving a power conversion efficiency of 7.12% [82]. The TiO_2_ NPs form a high-porosity 3D structure that increases dye adsorption due to the high surface area. Since then, scientists explored different approaches to increase conversion efficiency, and TiO_2_ has become one of the most often studied semiconductor materials in DSSCs, primarily due to its low costs, availability, and high stability against photocorrosion. One factor limiting the DSSCs efficiency is significant electron loss, which is attributed to increased electron recombination probability at grain boundaries between NPs [76]. In this process, electrons are found to be scattered due to structural defects and defects at the interface between the crystalline particles. This leads to a shorter electron lifetime, resulting in lower charge mobility and lower current density registered for the conducted reaction [81].

The use of a titania nanotube array—grown directly on Ti substrate by anodizing—was proposed to improve the performance of TiO_2_-based DSSCs [58,59,75]. The TiO_2_ nanotube layers possess highly ordered, vertically aligned 1D channels with a high aspect ratio for electron transport, in which the charge recombination is expected to be reduced, as compared to the TiO_2_ NP layer [62]. The longer electron diffusion lengths, more efficient light scattering, and shorter electron transport time constants all mean that the light-harvesting efficiencies of DSSCs based on vertically grown TiO_2_ nanotube arrays are higher than those found for DSSCs incorporating TiO_2_ nanoparticles (e.g., commercially available Degussa P25) [60,61]. The increased electron lifetime—estimated using open circuit voltage decay and electrochemical impedance spectroscopy (EIS) measurements—was observed for the TiO_2_ nanotube arrays as compared to TiO_2_ NP layers [20,62]. Indeed, the tube geometry also influences the electron lifetime [21,76]. Moreover, the nanotube arrays with 50–100 nm diameter might be beneficial for deep penetration of liquid electrolytes. Two comprehensive literature overview articles by Roy et al. [76] and Hou et al. [61] contain more information on the photovoltaic performance of the DSSCs constructed using TNT array layers on Ti by the anodizing process.

Initially, the TiO_2_ nanotube arrays obtained by anodizing were used directly with the residual Ti substrate layer in the dye-sensitized solar cells. The TNT/Ti is opaque, so the cell construction requires back-side illumination [75,77]. This solution leads to lower performance and diminished efficiency because of light absorption in the iodine electrolyte, and reflection at the Pt counter electrode [60,62,75]. Paulose et al. [75] reported a conversion efficiency of 4.24% for DSSCs with the highly ordered TNT/Ti films sensitized by N719 dye under AM 1.5 sunlight source.

The overall conversion efficiency of the back-side illuminated dye-sensitized TiO_2_ nanotubes-based solar cells remained relatively low. For this reason, several approaches have been proposed to construct front-side illuminated DSSCs, which exhibit improved *η* values [14,62]. One strategy involves producing a photoanode by completely anodizing a thin Ti layer sputtered directly on fluorine-doped tin oxide glass [60,63,64,75]. Such an approach led to low *η* values, and obtaining a thick TNT array layer with good adhesion to FTO proved to be difficult and expensive [62]. An interesting approach was proposed by Park and Chun [65]. The authors prepared TNT layers by completing Ti anodizing, grounding them into TNT particles, and using them in DSSC photoanode as a replacement for TiO_2_ nanoparticles. The measured DSSCs conversion efficiency under AM 1.5 illumination was approximately 5.03%. Joseph et al. [66] prepared DSSCs using TiO_2_ nanotubes obtained by powdering the TNT membranes detached from the Ti substrate. The authors revealed that the DSSC performance depended on the counter electrode employed during Ti substrate anodizing. The highest *η* was observed for Pt cathode (6.76%), while the energy conversion efficiency of over 6% was measured for charcoal, graphite pencil, and iron cathodes.

**Figure 5:**
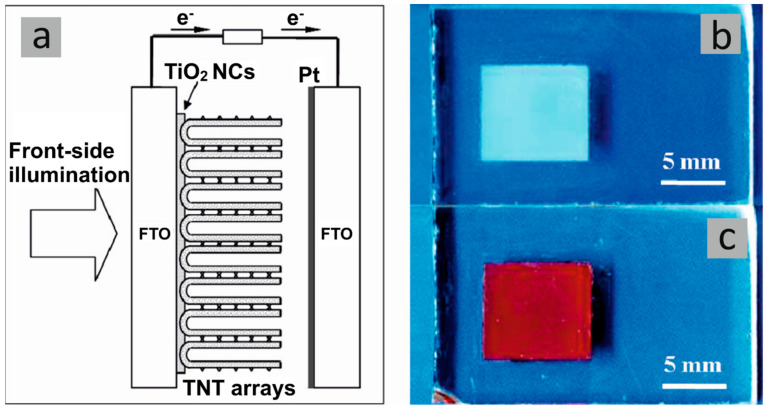
(**a**) Scheme of front-side illuminated DSSC fabricated using free-standing TNT layer. The photos of TNT (**b**) before and (**c**) after dye sensitization. Reprinted in part with permission from [62]. Copyright © 2009, American Chemical Society.

Alternatively, it was proposed to detach a free-standing TNT array layer from the Ti substrate and fix it onto FTO, using a binding interlayer. The schematic illustration of a dye-sensitized solar cell constructed using a free-standing TNT layer, as well as the images of the photoanode prior to and post-dye-sensitization, are shown in Figure 5 [62].

Optimization of the steps required for producing a free-standing TNT array layer on a DSSC photoanode has been the subject of many scientific papers. Firstly, the TNT layer has to be detached from the titanium substrate after anodizing. It is important, that the TNT layer has to be lifted in one flat piece, as cracks or other architectural voids negatively affect the light-harvesting characteristics of DSSCs [27,67]. Secondly, the crystalline structure of an as-anodized TNT array is amorphous; therefore, it requires annealing to transform it into a photo-active phase, anatase. The annealing temperature and the heating rate have been shown to affect the *η* of DSSCs [27,68]. Finally, the detached and annealed TNT layer has to be fixed to the FTO. For this purpose, different binders have been used. It is important to note that the TNT arrays were also used in solid-state dye-sensitized solar cells [107].

General techniques for material characterization of dye-sensitized solar cell components are electron microscopy, X-ray diffraction, X-ray photoelectron spectroscopy, UV-Vis, IPCE, scanning tunneling microscopy (STM), Raman, and FTIR spectroscopies. The information provided by the techniques mentioned above in the context of TNT membrane application in DSSC and in general, regarding morphology and nanostructure dimensions (SEM); crystalline phase (TEM, XRD); lattice spacing (TEM, SAED, XRD); elemental composition (SEM/TEM/EDX, XPS); optical properties/light harvesting efficiency (UV-Vis, IPCE); band gap (DRS, IPCE, UPS); dye coverage (UV-Vis); the absorption mode of the dyes on different crystalline surfaces of the material (STM, XPS, Raman, and FT-IR spectroscopies). Additionally, electrochemical methods, such as current-voltage, cyclic voltammetry, and electrochemical impedance spectroscopy, allow the study of charge transfer resistance and interfacial processes and are commonly utilized to characterize and analyze dye-sensitized solar cells (DSSC). To evaluate the surface area of newly tested materials for DSSC a BET method is considered [135].

#### 3.4.1. Methods of TNT Array Detachment Used for Photoanode Preparation

So far, multiple methods have been proposed to lift off of the TiO_2_ nanotube array layer from the Ti substrate during the DSSC preparation: chemical etching by immersion in 0.1 M aqueous HCl for 1 h [69]; detachment during repeated rinsing with ethanol and water [20,70,71,72]; detachment of amorphous TNT layer by ultrasonic vibration [60]; self-detachment by anodizing of annealed TNT/Ti layer [14,19,22,23,27,68,107]; washing in ethanol and immersion in 0.07 M HF [15,25,73]; and selective dissolution of amorphous TNT layer underneath annealed TNT layer in H_2_O_2_ solution [21,24,26,39,40,41,49,62,74]. Table 2 contains the best results reported for DSSCs constructed using the free-standing TNT layers, including the detachment method.

Park et al. [69] reported that for a free-standing 35 µm-thick TNT layer detached from the Ti by immersion in 0.1 M HCl solution, and annealed at 500 °C for 30 min in air, the DSSC exhibited a short circuit current of 16.8 mA cm^−2^, open circuit voltage of 0.733 V, fill factor of 62%, and overall conversion efficiency of 7.6%. It is important to point out that the annealing of the TNT layer post-detachment can lead to undesired cracking or curling of the lifted layer. To overcome such an issue, Chen and Xu employed the two-step anodizing approach to form a duplex crystalline-amorphous TNT array [62]. The TNT layer formed during the first anodizing was annealed and subsequently anodizing again in order to produce an amorphous layer at the interface of crystalline TNT and titanium substrate. The amorphous layer can be preferentially etched away by soaking it in a 10% H_2_O_2_ solution for 12 h. As reported, this approach allowed the formation of a crack-free TNT layer; however, it was highly time-consuming. The TiO_2_ 25 µm-thick layer DSSC exhibited improved performance (energy conversion efficiency 5.5%) compared to back-side illuminated DSSC and front-illuminated DSSC composed of TiO_2_ nanoparticle layer [62]. Other authors obtained similar approximately 5–6% efficiencies for the TNT layers detached in the same manner [26,40,49,74,78]. Interestingly, when the TiO_2_ quantum dot (QDs) blocking layers were inserted between conductive glasses and TNT membranes in the photoelectrodes, the resulting DSSCs power conversion efficiency reached 8.43% (it is 35.75% enhancement when compared to the sample without TiO_2_ QD) [24].

**Table 2:** The best results of photovoltaic performance of DSSCs, constructed using free-standing TNT arrays.

Anodizing Parameters	TNT Layer Detachment	TNT Layer Morphology			DSSC Measurement									Ref.
		Thickness (μm)	Tube Diameter (nm)	Wall Thickness (nm)	Photoanode Preparation	Electrolyte	Measurement Conditions		Results					
							Active Area (cm^2^)	Light Source	Short-Circuit Current Density (mA cm^−2^)	Open-Circuit Voltage (V)	Fill Factor (%)	Energy Conversion Efficiency (%)	Dye Loading (10^−8^ M cm^−2^)	
0.25 wt% NH_4_F, 2 vol% H_2_O, in EG 1st: 60 V	- Amorphous layer chemical dissolution in 0.1 M HCl for 1 h	35	~130 nm	~15	- Binded onto FTO (treated with 40 mM TiCl_4_) with 100 mM Ti-isopropoxide - Annealed at 500 °C for 30 min in air - Sensitized with N719 dye	0.6 M butylmethylimidazolium iodide, 0.03 M I_2_, 0.1 M guanidinium thiocyanate, 0.5 M 4-tert-butylpyridine, in ACN and valeronitrile (85:15, vol/vol)	0.03–0.15	AM 1.5	16.8	0.733	62	7.6	-	[69]
0.25 wt% NH_4_F, in EG 1st: 50 V, 24 h	- Annealing at 330–500 °C for 0.5–2 h in oxygen - Anodizing at 12 V for 3–8 h - Amorphous layer chemical dissolution in 10% H_2_O_2_ for 12 h	~25	~50 nm	-	- Annealed at 500 °C for 2 h - Binded onto FTO coated with layer (3 µm) of TiO_2_ NPs paste - Sintered at 450 °C for 30 min - Treated with 0.05 M TiCl_4_ - Sintered at 450 °C for 30 min - Sensitized with N719 dye	0.05 M LiI, 0.05 M I_2_, 0.6 M PMII, 0.5 M 4-terbutylpyridine in 3-methoxyproprionitrile	0.20	Xe lamp	12.4	0.701	63.3	5.5	-	[62]
0.25 wt% NH_4_F, 0.75 wt% H_2_O, in EG + PEG (4:1, *v*/*v*) 1st: 50 V, 4 h	- Mechanical detachment/ultrasonic vibration in H_2_O	20.8	99	27	- Binded onto FTO with drop of TiO_2_ sol containing Ti(OBu)_4_ and PEG - Annealed at 200 °C for 1 h and at 500 °C for 3 h - Treated with 0.04 M TiCl_4_, - Sintered at 500 °C for 30 min - Sensitized with N719 dye	0.03 M I_2_, 0.6 M PMII, 0.10 M guanidinium thiocyanate, 0.5 M *tert*butylpyridine, in ACN/valeronitrile (85:15, vol/vol)	-	AM 1.5	15.46	0.814	64.1	8.070	15.8	[60]
0.5 wt% NH_4_F, 3 wt% H_2_O_2_, in EG 1st: 80 V, 12 h, 20 °C	- Amorphous layer chemical dissolution in 33 wt% H_2_O_2_ for 20 s *Barrier layer removed in 0.5 wt% oxalic acid at 40 °C for 16 h	~70	Inner ~140	~50	- Annealed at 450 °C for 30 min - Adhered to FTO coated (doctor blade) with a TiO_2_ NPs paste (3 µm) in H_2_O and PEG formed by sol-gel method - Annealed at 450 °C for 30 min - Treated in 0.05 M TiCl_4_ and annealed - Sensitized with N719 dye	0.5 M LiI, 0.05 M I_2_, 0.5 M tert-butylpyridine in dry ACN	0.25	AM 1.5	TNT array with barrier layer					[78]
									11.7	0.714	63	5.3	15.9	
									TNT array without barrier layer					
									18.5	0.77	64	9.1	15.2	
0.2 M NH_4_F, 0.01 M H_3_PO_4_, 2.2 wt/vol% H_2_O, in EG 1st: 60 V, 5 h	- Annealing at 450 °C for 30 min - Anodizing at 60 V for 30 min - Amorphous layer chemical dissolution in 0.07 M HF and shaking	22–23	-	-	- Transferred onto FTO screen-Printed with NPs TiO_2_ paste (2.5–2.7 µm) - Freeze-dried at −20 °C - Annealed at 450 °C for 30 min. - Treated with 20 mM TiCl_4_ for 1 h - Sintered at 450 °C for 30 min - Sensitized with N719	0.6 M 1-propyl-2,3-dimethylimidazolium iodide, 0.05 M I_2_, 0.1 M LiI, 0.1 M guanidine thiocyanate, 0.5 M *tert*-butylpyridine, in ACN*/*valeronitrile (1:1, vol/vol)	0.16	AM 1.5	**OED open-ended down (facing NP layer)**					[73]
									16.3	0.700	53	6.12	-	
									**CED closed-ended down (facing NP layer)**					
									9.68	0.630	61	3.75	-	
0.5 wt% NH_4_F, 3 vol% H_2_O, in EG 1st: 60 V, 0.5 h Removed by ultrasonication in DI 2nd: 60 V, 1 h	- Annealing at 200 or 400 °C for 30 min - Anodizing at 60 V, for 1 h at 30 °C - Self-detachment during 3rd anodizing *Tubes opened or closed, depending on the annealing temp.	23.8	-	-	- Transferred onto FTO spin-coated with P25 TiO_2_ NPs paste (3 vol.% acetic acid solution) - Sintered at 450 °C, 3 h - Sensitized with N719 dye	DMPII/LiI/I_2_/TBP/ GuSCN in ACN	-	AM 1.5	**TNT with nanotubes opened on both sides (200 °C)**					[14]
									10.65	0.70	70	5.32	-	
									**TNT with nanotubes opened on one side (400 °C)**					
									10.32	0.71	62	4.52	-	
0.5 wt% NH_4_F, 3 vol% H_2_O, in EG 1st: 60 V, 30 min, RT Removed by ultrasonication 2nd: 60 V, 2 h	- Annealing at 250–650 °C for 2 h in air - Anodizing at 60 V for 10–30 min at 45 °C - Self-detachment during 3rd anodizing	7.86	-	-	- Transferred onto FTO CBU (closed Bottom up, facing P25 NPs), pre-treated with TiCl_4_ and printed with P25 paste (in ethanol, acetic acid, PEG and ethocel) by doctor-blade technique - Annealed at 125–450 °C - Treated with 40 mM TiCl_4_ - Sensitized in N719	DHS-E23	0.64	AM 1.5	15.60	0.76	64	7.62	25.47	[19]
0.6 wt% NH_4_F, 2 vol% H_2_O, in EG 1st: 50 V, 4 h	- Drying at 200 °C for 20 min - Annealing at 480 °C for 40 min - Anodizing at 70 V for 1 h - Self-detachement during 2nd anodizing	16	-	-	- Transferred in IPA onto FTO spin-coated with Ti-isopropoxide (in IPA, Triton X-100, acetic acid) and annealed at 450 °C for 30 min - Dropped with Ti-isopropoxide solution - Dried at 200 °C for 15 min - Annealed at 480 °C for 30 min - Treated with 40 mM TiCl_4_ and sintered at 480 °C for 30 min	Solid electrolyte: 0.2 mM triethylammonium thiocyanate in a mixture of saturated Ni(SCN)2, α-CuSCN and β-CuSCN propyl-sulfide (PS) solutions (vol. ratio 1:1:10)	0.5	AM 1.5	-	-	-	6.3		[107]
0.5 wt% NH_4_F, 2.5 vol% H_2_O, in EG 1st: 60 V, 2 h	- Mechanical detachment/solvent evaporation; rinsing in DI and ethanol	12	Outer 120		- Binded onto FTO with TiO_2_ sol made with Ti-isopropoxide and Tween 20 - Annealed at 450 °C for 1 h - Treated with 0.05 M TiCl_4_, - Annealed at 450 °C for 30 min - Sensitized with N719 dye	Iodolyte AN 50	0.22	AM 1.5	14.61	0.66	63	6.07	-	[70]
0.5 wt% NH_4_, 2.5 vol% H_2_O, in EG 1st: 60 V, 30 min Removed by ultrasonication in ACN 2nd: 60 V, 4 h	- Mechanical detachment/solvent evaporation; rinsing in DI and ethanol	30	Outer 120	20	- Transferred onto FTO coated with TiO_2_ NPs sol (prepared using Ti-isopropoxide, glacial acetic acid and Tween 20) - Annealed at 450 °C for 1 h - Treated with 50 mM TiCl_4_ - Annealed at 450 °C for 30 min - Sensitized with N719 dye	Iodolyte AN 50	-	AM 1.5	17.47	0.677	64	7.56	-	[20]
0.5 wt.% NH_4_F, 3 vol.% H_2_O, in EG 1st: 60 V, 20 °C	- Annealing at 400 °C - Anodizing at 60 V for 1–2 h at 35 °C - Self-detachment during 2nd anodizing	13	-	10–20	- Annealed at 700 °C for 2 h - Transferred onto FTO coated with anatase NPs layer (1 µm) by doctor-blade technique - Annealed at 400 °C for 2 h - Sensitized with N719 dye	1.0 M 1,2-dimethyl-3-propyl imidazolium iodide (DMPII), 0.12 M I_2_, 0.1 M LiI, 0.5 M 4-tertbutylpyridine (TBP) in 3-methoxypropionitrile (MPN)	0.16	AM 1.5	16.02	0.76	64	7.81	9.7	[68]
0.5 wt% NH_4_F, 2 vol% H_2_O, in EG 1st: 60 V, 8 h	- Annealing at 450 °C for 2 h - Anodizing at 15 V for 4 h - Mechanical delamination/solvent evaporation; rinsing in ethanol + mechanical vibration	32	100	-	- Transferred onto FTO spin-coated with 0.1 M Ti-isopropoxide in IPA - Dropped with 0.5 M Ti-isopropoxide - Freeze-dried at −20 °C - Sensitized with N719	0.5 M KI, 0.05 M I_2_, in EG	-	UV (368.1 nm) LED	-	-	-	10.6	-	[71]
0.5 wt% NH_4_F, 2 vol% H_2_O, in EG 1st: 40 V, 12 h, RT	- Annealing at 450 °C for 30 min - Anodizing for 3 h - Amorphous layer chemical dissolution in 35.5 wt% H_2_O_2_	15	~120	~ 20	- Transferred onto FTO spin-coated with P25 TiO_2_ NPs viscous paste layer (9 µm) - Sintered at 450 °C for 30 min - Sensitized with N719 dye	0.3 M LiI, 0.05 M I_2_, 0.6 M 1-propyl-3-methylimidazolium iodide, 0.5 M *tert*-butylpyridine in dry ACN	-	AM 1.5	15.88	0.65	61	6.32	4.43	[74]
0.08 M NH_4_F, 1.5 wt% H_2_O, in EG, 1st: 60 V, 60 min Removed 2nd: 40–60 V, 200 min	- Annealing at 450 °C for 120 min - Anodizing for 30 min - Amorphous layer chemical dissolution in 10% H_2_O_2_, 10 min	14	60 on tube top and 100 on bottom	-	- Transferred on ITO/polyethylene naphthalate (PEN) substrate coated with P25 NPs slurry layer (5 µm) by doctor-blade technique - Sensitized with N719 dye	0.05 M I_2_, 0.5 M LiI, 0.3 M DMPII and 0.5 M 4-TBP, in ACN solution	-	AM 1.5	8.17	0.705	66.1	3.78	-	[21]
0.5 wt% NH_4_F, 3.0 vol% H_2_O, in EG 1st: 60 V, 30 min, RT Removed by ultrasonication 2nd: 60 V, 2 h	- Annealing at 450 °C for 2 h - Anodizing at 45 °C - Self-detachment during 3rd anodizing	10.88 ± 0.06	Inner 98, outer 147	-	- Annealed at 250 °C and 450 °C for 1 h - Transferred onto FTO pre-treated with 40 mM TiCl_4_ solution and coated with P25 paste (ethanol, acetic acid, PEG, ethocel) by doctor-blade technique - Annealed at 125–450 °C - Treated with 40 mM TiCl_4_ for 30 min - Annealed at 450 °C for 30 min - Sensitized in N719 dye	DHS-E23	0.64	AM 1.5	15.64 ± 0.15	0.759 ± 0.015	64 ± 2	7.64 ± 0.24	25.47 ± 1.62	[22]
0.15 M NH_4_F, 3 vol% H_2_O, in EG 1st: 60 V, 1 h, RT	- Annealing at 350 °C for 1 h - Anodizing at 60 V for 1 h at RT - Amorphous layer chemical dissolution in 0.07 M HF at 30 °C	Approximately 20	-	-	- Transferred on FTO coated with 2 µm-thick layer of TiO_2_ NPs paste by doctor-blade technique (tube top down toward NPs) - Annealed at 500 °C for 1 h (30 °C min^−1^) - Sensitized with D719 dye	Io-li-tec, ES-0004	0.2	AM 1.5	22.60	0.79	55	9.79	12.5	[15]
0.5 wt% NH_4_F, 3 vol% H_2_O, in EG 1st: 60 V, 1 h, RT Removed by ultrasonication 2nd: 60 V, 1 h	- Annealing at 450 °C - Anodizing at 30 °C - Self-detachment during 3rd anodizing	19	100		- Transferred on ITO/PET spin-coated with P25 NPs solution (in acetic acid and ammonia) - Laser-sintered (1064 nm, power 2.5 W) - Sensitized with the N719 dye	DMPII/LiI/I_2_/TBP/GuSCN in 3-methoxypropionitrile	-	AM 1.5	9.35	0.73	68	4.65	-	[23]
0.3 wt% NH_4_F, 2 wt% H_2_O, in EG 1st: 50 V, 1 h, RT Removed by ultrasonication 2nd: 50 V, 3 h	- Annealing at 450 °C for 2 h - Anodizing at 80 V for 5 min - Amorphous layer chemical Dissolution in 30 wt% H_2_O_2_ for 1 h	16.7	Inner 60, outer 100	20	- Transferred onto FTO spin-coated with TiO_2_ QDs toluene solution, sintered at 450 °C for 30 min and subsequently clung with 20 nm TiO_2_ NPs as cementing agent - Annealed at 450 °C for 30 min - Treated with 0.05 M TiCl_4_ - Sintered at 450 °C for 30 min - Sensitized with N719 dye	0.4 M sodium iodide, 0.1 M tetrabutyl ammonium iodide, 0.5 M 4-tert-butylpyridine, 0.05 M iodine, in ACN	0.12	AM 1.5	16.28	0.766	67.6	8.43	-	[24]
0.8 wt% NH_4_F, 2 vol% H_2_O, in EG 1st: 60 V, 1 h, 25 °C	- Annealing at 500 °C for 1 h at 30 V for 10 min - Amorphous layer chemical dissolution in 10% H_2_O_2_ *Barrier layer removed by Ar+ bombardment	~22	-	-	- Transferred on FTO spin-coated with 5 wt % Ti-di-isopropoxide bis(acetylacetonate) in butanol, annealed at 500 °C for 1 h, and coated with TiO_2_ paste - Annealed at 500 °C for 1 h - Treated with 10 mM TiCl_4_ - Annealed at 500 °C for 1 h - Sensitized with N719 dye	0.7 M 1-butyl-3-methyl-imidazolium iodide (BMII), 0.03 M I2, 0.1 M guanidium thiocyanate (GSCN), 0.5 M 4-tertbutylpyridine (TBP), in ACN/valeronitrile (85:15, vol/vol).	0.25	AM 1.5	11.56	0.79	67	6.12	-	[41]
0.1 M NH_4_F, 2 vol% H_2_O, in EG 1st: 60 V, 45 min Removed by ultrasonication 2nd: 2 h	- Annealing at 250 °C for 1 h in air - Anodizing - Amorphous layer chemical dissolution in aqueous 0.07 M HF	-	Inner ~110	~17	- Transferred onto FTO coated with TiO_2_ NPs paste by doctor-blade technique - Annealed at 450 °C for 1 h - Treated with 0.2 M TiCl_4_ - Annealed at 450 °C for 15 min - Sensitized with N719 dye	Electrolyte containing I/I^−3^ redox	-	AM 1.5	11.3	0.72	65	5.2	-	[25]
0.8 wt% NH_4_F, 2 vol.% H_2_O, in EG 1st: 60 V, 2 h, 25 °C	- Annealing at 500 °C for 30 min - Anodizing at 30 V for 10 min - Amorphous layer chemical Dissolution in 10% H_2_O_2_ *Barrier layer removed by Ar+ ion milling	18	100	-	- Transferred onto FTO coated with TiO_2_ NPs paste - Sintered at 500 °C for 1 h - Treated with 0.01 M TiCl_4_ - Sintered at 500 °C for 1 h - Sensitized with N719 dye	0.7 M 1-butyl-3-methyl-imidazolium iodide (BMII), 0.03 M I_2_, 0.1 M guanidium thiocyanate (GSCN), and 0.5 M 4-tertbutylpyridine (TBP), in ACN/valeronitrile (85:15, vol/vol)	-	AM 1.5	TNT array without barrier layer					[40]
									9.12	0.81	73	5.39	15.0	
									TNT with barrier layer					
									7.87	0.80	71	4.47	13.8	
0.33 g NH_4_F, 2 mL H_2_O, 98 mL EG 1st: 40 V,	- Annealing at 450 °C for 1 h - Mechanical detachment/solvent evaporation; rinsing in ethanol and H_2_O	-	Inner 40–60	30–60	- Transferred to FTO coated with TiO_2_ NPs by doctor-blade technique - Calcinated 450 °C for 30 min - Sensitized with N719 dye	Iodolyte	0.16	AM 1.5	12.71	0.77	73.85	7.24	-	[72]
0.5 wt% NH_4_F, 2.0 wt% H_2_O, in EG 1st: 60 V, 1 h Removed by ultrasonication 2nd: 60 V, 2 h	- Annealing at 250 °C for 30 min - Anodizing at 100 V - Amorphous layer chemical dissolution in 30 wt% H_2_O_2_	18	Inner 100, outer 170	-	- Transferred onto FTO coated with layer of TiO_2_ paste - AC-electrodeposited with TiO_2_ NPs - Sintered at 500 °C for 2 h - Sensitized with N719 dye	ELT-ACN-I: Standard Iodine Based Electrolyte	0.25	AM 1.5	14.73	0.69	61.0	6.12	-	[26]
0.3 wt%, NH_4_F, 2 vol% H_2_O, in EG, 1st: 60 V, 4 h, 27 °C	- Mechanical detachment/solvent evaporation; rinsing in water *Barrier layer removed by ion-beam etching	24	150	-	- Adhered on FTO with TiO_2_ sol - Annealed at 400 °C for 1 h - Sensitized with N719 dye	Iodolyte AN-50	0.25	-	14.40	0.594	40.7	3.48	-	[42]
0.15 M NH_4_F, 3 vol% H_2_O, in EG 1st: 60 V, 4 h, 30 °C Removed by ultrasonication 2nd: 60 V, 4 h, 30 °C	- Annealing at 520 °C for 6 h - Anodizing at 80 V for 20–40 min - Self-detachment during 3rd anodizing *Barrier layer etching by HF vapour for 60 min	26.60	-	-	- Transferred in IPA onto FTO coated with Ti-isopropoxide solution (in IPA, Triton X, acetic acid) and annealed at 500 °C for 30 min - Dropped with Ti-isopropoxide solution and annealed at 500 °C for 30 min - Treated with 40 mM TiCl_4_ - Annealed at 500 °C for 30 min - Sensitized with N719 dye	Irasol	-	AM 1.5	6.75	0.684	60	2.76	-	[27]
0.8 wt% NH_4_F, 2 vol% H_2_O, in EG 1st: 60 V, 2 h, 25 °C	- Annealing at 500 °C for 30 min - Anodizing at 30 V for 15 min - Amorphous layer chemical dissolution in 10% H_2_O_2_ *Barrier layer removed by Ar^+^ ion milling	18	100	-	- Transferred onto FTO spin-coated with Ti-isopropoxide bis(acetylacetonate) in butanol (5 wt%) - Annealed at 500 °C for 1 h - Treated with TiCl_4_ - Annealed at 500 °C for 1 h - Sensitized with N719 dye	0.7 M 1-butyl-3-methyl-imidazolium iodide, 0.03 M I_2_, 0.1 M guanidium thiocyanate, 0.5 M 4-tertbutylpyridine, in ACN/valeronitrile (85:15, vol/vol)	-	AM 1.5	10.64	0.795	69.4	5.87 ± 0.47		[39]
0.8 wt% NH_4_F, 2 vol% H_2_O, in EG 1st: 60 V, 2 h, 25 °C	- Annealing at 500 °C for 30 min - Anodizing at 30 V for 15 min - Amorphous layer chemical dissolution in 10% H_2_O_2_	18	100	-	- Transferred onto FTO spin-coated with Ti-isopropoxide bis(acetylacetonate) in butanol (5 wt%) and TiO_2_ paste by doctor-blade technique - Annealed at 500 °C for 1 h - Treated with TiCl_4_ - Annealed at 500 °C for 1 h - Sensitized with N719 dye	0.7 M 1-butyl-3-methyl-imidazolium iodide, 0.03 M I_2_, 0.1 M guanidium thiocyanate, 0.5 M 4-tertbutylpyridine, in ACN/valeronitrile (85:15, vol/vol)	-	AM 1.5	10.27	0.764	65.9	5.17	-	[49]

Wang et al. [71] reported a similar approach, where detachment of the duplex crystalline/amorphous layer occurred due to repeated rinsing with ethanol. The highest anatase content of 92% as well as energy conversion efficiency of 10.6% under UV illumination (368.1 nm), was observed for DSSCs containing samples re-anodizing at 15 V for 4 h. The free-standing TNT array-based DSSCs, obtained by anodizing at 40 V and detached using the same method, showed an energy conversion efficiency value of 7.24% under AM 1.5 illumination [72]. Detachment by rinsing with ethanol and water could also be achieved for amorphous TNT layers after one-step anodizing (12 µm) and two-step anodizing (30 µm), leading to the DSSCs efficiency at 6.07% and 7.56%, respectively [20,70].

Dubey et al. [73] achieved detachment of the duplex crystalline/amorphous TNT layer (22–23 µm) by rinsing with ethanol and subsequent immersion in 0.07 M HF solution (amorphous TNTa dissolution). The resulting DSSCs showed an efficiency of 3.75%. This method, although fast, required subsequent washing to neutralize the acid used. Mohammadpour et al. [15] reported an energy conversion efficiency of 9.79% for DSSCs constructed using a approximately 20 µm-thick TNT layer detached using a 0.07 M HF solution. It is important to point out that aggressive methods of TNT array detachment would often lead to undesired fracturing or damage to the TNT array [107], especially after prolonged storage in solution or complete drying [73].

Lei et al. [60] detached amorphous TNT array from Ti substrate through ultrasonic vibration. Although the number of steps in the synthetic procedure was limited, the power conversion efficiency for a 20.8 μm-long TiO_2_ nanotubes layer was 8.07%, while an open-circuit voltage, a short-circuit current, and a fill factor were 0.814, 15.46 mA cm^−2^, and 64.1%, respectively.

Finally, the TNT duplex layer self-detachment could also occur during third anodization, leading to DSSCs *η* value of 4.52% [14], 7.62% [19], 7.64% [22], or during second anodization with DSSCs power conversion efficiency value of 7.81% [68].

In the aforementioned reports, the nanotube bottoms were closed on one side by a so-called barrier layer. It was suggested that the barrier layer could potentially cause absorption of near-UV light, reflect front surface light, and block diffusion of the electrolyte or dye [78]. Hence, various methods were implemented to remove the barrier layer from the free-standing TNT layer to prepare DSSCs photoanode, which will be the subject of further discussion.

It should also be noted that the performance of free-standing TNT array-based DSSCs depends on the length of the nanotubes [62,69], nanotube morphology [76], the orientation of the TNT layer structure inside the photoanode, and the removal of the barrier layer [27]. Moreover, fixing the TNT array onto the FTO surface was solved using different strategies, often including applying additional layers of TiO_2_ nanoparticles, which would additionally affect the performance of the DSSCs [62]. 

Thus, considering the cited scientific results, the front-side illumination, optimization in anodizing conditions and the detachment process is a course of action to improve DSSC efficiency.

#### 3.4.2. Influence of the TNT Array Thickness on DSSC Performance

As described above, the length of the TiO_2_ nanotubes increases with the duration of the anodizing. Park et al. [69] observed that the photocurrent of the DSSCs increased linearly with the thickness of the TiO_2_ layer up to 35 µm. Chen and Xu observed an increase in the short-circuit current density for the 15–25 µm-long TNT from 11.0 mA cm^−2^ to 12.4 mA cm^−2^, and a significant decrease in *J_SC_* and *V_OC_* for 37 µm-long TNT-based DSSC [62]. Scientists have explained the abovementioned experimental results by referring to the work of Jennings et al. [59] In their report, the authors stated that for a 20 µm thick TNT layer, the peak of IPCE was 90% and the collection efficiency for photoinjected electrons was almost 100% under short circuit conditions. The IPCE spectrum measured by scientists was in good agreement with the value calculated from the dye loading of the TiO_2_ nanotubes layer determined by UV-Vis spectroscopy. Therefore, Chen and Xu proposed, that the dye reached saturation for 25 µm-long TiO_2_ nanotubes. For thinner layers, the current density and efficiency would increase with the number of adsorbed dye molecules, while for thicker layers, an increase in the surface area and adsorbed dye molecules would produce more recombination centers. Lei et al. [60] reported the best results for DSSCs with 20.8 μm-long TNT. Li et al. [107] showed, that the highest DSSC efficiency of 6.3% was obtained for a sample with a TNT thickness of 16 µm, obtained by 4 h of long anodizing; however, the open circuit voltage and fill factor were independent of the length of the TiO_2_ nanotubes. Results presented by Huang et al. [22] revealed that the best energy conversion efficiency of 7.64% was obtained for samples with a TNT thickness of 10.88 µm. Increased length of TNTs with more adsorbed dye molecules would not improve light harvesting, as the thickness of this layer would hinder both light penetration and carrier transport. Lamberti et al. [20] showed increasing *η* with the increasing TNT thickness in a range of 12–30 µM. According to Lan et al. [24], there is a point in the thickness of TNT array, where two converse effects are balanced, leading to maximum short-circuit current and energy conversion efficiency. Experimental results have shown that increasing the length of TiO_2_ nanotubes can enhance their diffuse reflectance ability and light absorption, but it can also lead to unwanted recombination reactions. Therefore, the authors reported the best results for samples with a TNT thickness of 16.7 µm, obtained by a 3 h anodizing process [24].

#### 3.4.3. Result of TNT Array Configuration and Barrier Layer Removal

The TNT layer detached from the Ti substrate is asymmetric. The top side with open nanotubes is rough, while the bottom side is smoother, with the nanotubes closed by caps of the so-called barrier layer. Different groups reported conflicting information on the effect of the TNT layer placement on the photoanode performance in DSSCs [15,22,27]. For example, Dubey et al. [73] showed better adhesion between the TNT layer and TiO_2_ layer when the top side was placed toward the FTO, leading to better energy conversion efficiency, as compared to TNT placed with the bottom side towards FTO. In later studies, the converse TNT layer placement was reported [25]. Peighambardoust et al. [27] studied the effect of the orientation of TNT layers doped with non-metal N ions. DSSC with a CED (closed-end down toward FTO) TNT configuration gave a higher short-circuit current density than the OED (opened-end down) one. The authors reported localized lifting of the TNT layer from the FTO in OED orientation, leading to hindered electron transfer. In the OED configuration, the dye loading was lower than in the CED orientation, as the presence of the barrier layer inhibited dye solution penetration.

Conversely, the barrier layer removal effect on the DSSCs is well-documented in the literature, improving the light-harvesting and electron collection efficiency. The barrier layer hinders the flow of the electrons from the TiO_2_ conduction band and FTO. Lin et al. [78] compared the performance of DSSCs formed using TNT with closed and opened tubes. They reported that TNT with the barrier layer removed by treatment in 0.5 wt% oxalic acid solution achieved an energy conversion efficiency of 9.1%, while TNT with closed nanotubes exhibited an efficiency of 5.3%. The same effect was reported by other groups, which used different techniques of TNT caps removal, such as ion milling [40,42], adjusting the annealing temperature [14], or etching with HF vapor [27].

Peighambardoust et al. [27] reported a notable enhancement of the photovoltaic performance of DSSCs containing a through-hole morphology of N-doped TNT membrane. The EIS measurements revealed that in through-hole morphology, the highest capacitance and the lowest resistance could be observed as a result of better dye-loading when compared to CED and OED orientation. A high dye-loading density leads to a higher electron density at the conduction band, increasing the Fermi levels in TiO_2_, a higher *V_OC_*, and consequently a higher energy conversion efficiency. Importantly, no obvious change in electron lifetime was observed for DSSC with different configurations [27].

#### 3.4.4. Effect of the Annealing Temperature and Heating Rate

**Figure 6:**
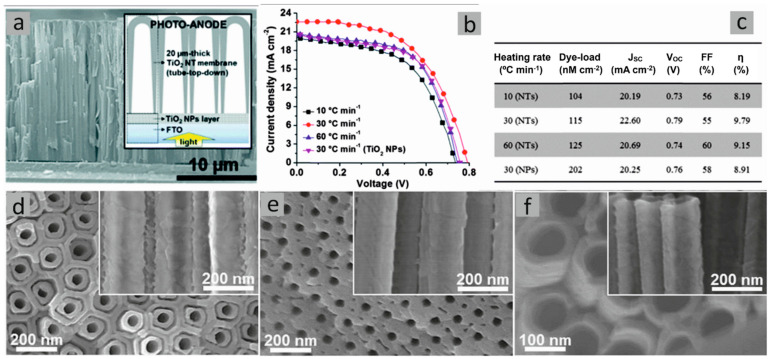
(**a**) Cross-sectional SEM image of a 20 µm-thick TNT membrane. Inset: a scheme of the photoanode with OED configuration. (**b**) J–V curves and (**c**) a summary of the photovoltaic performance of DSSCs constructed using TNT layers annealed at selected heating rates. Top-view and cross-section SEM images of TNT membranes annealed in air at 500 °C at a heating rate of (**d**) 10, (**e**) 30, and (**f**) 60 °C min^−1^. Reprinted in part with permission from [15]. Copyright © 2015, Royal Society of Chemistry.

The amorphous structure of the free-standing TNT layer needs to undergo annealing for photoactive anatase and/or rutile phase transformation. Multiple authors have reported the effect of the annealing temperature on the DSSC efficiency. Lin et al. [68] showed the best DSSC performance for the TNT array annealed at 700 °C. Peighambardoust et al. [27] concluded that by increasing the annealing temperature from 480 to 520 °C, the cell efficiency was improved by up to 70%. However, in other studies, the annealing temperature was optimized to approximately 450 °C, as it led to a complete transformation of the amorphous TNT array into an anatase phase and roughening of the nanotube surface due to crystallite formation [20]. This phenomenon would affect the electron transport along the tubes, but it can also further increase the surface area and, thus the dye loading. In the research performed by Mohammadpour et al. [15] cell efficiency (*η*) of 8.19% was reported for samples annealed at 500 °C. The authors suggested that the annealing temperature and the heating rate would influence the DSSC’s performance, as shown in Figure 6. Fast annealing at the heating rate from 10 to 30 °C min^−1^ led to the formation of a robust, crack-free, and ‘‘single-walled’’ morphology, resulting in enhanced solar light conversion efficiency up to 9.79%. Conversely, slower annealing caused a relatively large number of cracks, which might be responsible for inducing charge carrier recombination and limiting the electron lifetime.

#### 3.4.5. Impact of a Binder

Fixing the free-standing TNT layer to the FTO appears to be the most challenging and tedious step in photoanode construction. The utilized approach has an important role in the constructed DSSC photovoltaic performance. Park et al. [69] used 100 mM Ti-isopropoxide to fix the TNT to FTO; however, the bonding achieved was weak. Lei et al. [60] utilized a toxic solution of Ti(OBu)_4_ and polyethylene glycol as a binder. Alternatively, the TiO_2_ bonding layer was prepared using TiO_2_ NPs paste [62,73,74] or TiO_2_ sol [52,70], followed by sintering under pressure to avoid the TNT layer curling and peeling. TiO_2_ NP paste is commonly applied by screen printing or the doctor-blade technique, which leads to an irreproducible layer thickness of 2.5–4.5 µm [73]. Such a layer’s thickness could influence the photoanode’s mechanical stability during sintering. Li et al. [107] showed, conversely to previous reports, [62,136] that over 96% of the efficiency originates from the TNT layer rather than the adhesion nanoparticle paste layer.

Hu et al. [74] demonstrated that DSSCs with superior properties were achieved when the TNT layer was adhered to FTO with commercially available TiO_2_ NPs (Degussa P25) layer compared to pure P25-DSSC and pure TNT-DSSC. This effect was explained by the increased amount of the adsorbed dye molecules on the double-layered films. The EIS measurements revealed that pure TNT-DSSC had the highest resistance due to poor contact between the TNT and FTO. Interestingly, a similar electron lifetime of ca 60 ms was observed for both pure TNT and double layers of TNT over Degussa P25. By using the TiO_2_ sol instead of TiO_2_ NP paste, Lamberti et al. [70] increased the electron lifetime to 177 ms at *V_OC_* by reducing the adhesion layer thickness, which lowered the electron recombination rate at the interfaces.

Peighambardoust et al. [27] suggested that to improve the TNT array adhesion, FTO should be firstly coated with Ti-isopropoxide, IPA, Triton X-100, and acetic acid solution (TTIP), followed by annealing at 500 °C for 30 min, which resulted in a 50 nm-thick anatase buffer layer. Such a layer would facilitate the TNT array adhesion and prevent direct charge recombination at the FTO/electrolyte interface.

#### 3.4.6. Strategies of TNT Layers Modification and Doping in DSSC

One of the factors limiting the TiO_2_ nanotube DSSC efficiency is the existence of bulk and surface trap sites. To further improve the properties of DSSC it is important to lower the density of trap states, which have hindering effects on the charge transport. This can be achieved by doping with different elements, e.g., N [27]. The N-doping of the TNT array was carried out by immersing the amorphous TNT/Ti samples in a 1 M NH_3_·H_2_O solution for 15 h, followed by annealing at 520 °C for 6 h. This led to an increase in the energy conversion efficiency of the DSSCs by up to 40% when compared to the undoped samples. The efficiency value of the DSSC using an N-doped TNT membrane with the barrier layer removed by etching in HF solution reached nearly 8% under AM 1.5 illumination [27]. The high efficiency was due to the two-open-ends configuration of the tubes, which resulted in higher dye loading and higher charge recombination suppression.

Peighambardoust et al. [27] also explored the effect of using blue TiO_2_ nanotube membranes in DSSCs. The blue TNT layers were obtained by cathodic electrochemical reductive polarization in 0.5 M Na_2_SO_4_ at a potential of −1.4 V for 10 min. As a result, Ti^3+^ states were generated. Despite the narrowing of the band gap, the resulting DSSC energy conversion efficiency was low because the self-doped TNT layer could not be heated up to more than 200 °C during dye-sensitization. As a result, fewer dye molecules were adsorbed on the TiO_2_ surface, decreasing the density of photo-excited electrons.

Chamanzadeh et al. [25] studied the light-harvesting performance of DSSCs with TNT layer modified with ZnO NPs using a sol-gel spin coating technique. The results obtained for *J_SC_*, *Voc*, and *FF* were 11.3 mA cm^−2^, 0.72 V, and 65%, respectively. The ZnO NPs layer over TiO_2_ nanotubes led to a power conversion efficiency of 5.2%. Further increase in the number of ZnO NP layers caused a decrease in the short-circuit current density due to the rise of trapping sites at the TiO_2_/ZnO interface and the formation of dye-Zn ion complexes zinc oxide is vulnerable to corrosion in acidic dye environments, leading to hindered dye absorption. An additional improvement of the DSSCs performance was achieved for TNT treated with TiCl_4_ before ZnO NPs coating deposition. The *V_OC_* was 0.82 V, the *J_SC_* was 16.1 mA cm^−2^, the *FF* was 63%, and the power conversion efficiency was 8.3%, due to the downward shift of the conduction band edge of the TiO_2_. The EIS measurements, intensity-modulated photovoltage, and photocurrent spectroscopies measurements revealed that the optimal combination of TiO_2_ nanotubes covered with ZnO NPs reduced electron recombination and improved transport pathways, leading to charge collection of 99%.

In a series of papers, Rho and co-workers modified TNT membranes with Ag NPs [47], Ag NPs and TiO_2_ particles [41], carbon [38], Ag NPs and carbon materials [48], TiO_2_ particles and carbon materials [40], Au NPs [49], and Au NPs and carbon materials [39]. The Ag NPs were deposited inside TiO_2_ nanotubes by photochemical reaction, and the Au NPs were electrochemically deposited, leading to increased overall energy conversion efficiency of DSSCs due to plasmonic effects (surface plasmon resonance along the nanotubes) and improved electron transport [39,47]. Doping with carbon, achieved by chemical vapor deposition [38], enhanced the light-harvesting performance of the DSSCs because of π-π conjugation, affecting the efficiency of electron transport [38,39,40]. Coating the TNT layers with large TiO_2_ particles also improved energy conversion efficiency, creating a light-scattering effect [41]. The highest *η* values of 7.24%, were reported for DSSCs containing TNT membranes with the barrier layer removed by ion milling and modified with Au NPs and carbon materials [39]. Indeed, combining two modification methods led to increased power conversion efficiency of up to 30%, when compared to unmodified TNT membranes-based DSSCs [40,48].

### 3.5. Quantum Dots Sensitized Solar Cells

Narrow-band semiconductor quantum dots (QDs) have recently been used as a sensitizer in solar cells (QDSSCs). The increased scientific attention given to QDSSCs is related to their superior properties, such as high extinction coefficient, tunable band gap, multiple exciton generation effects, and predicted maximum energy conversion efficiency 44% higher than for DSSCs [79]. The QDSSCs are constructed similarly to DSSCs. However, instead of a dye, the QDs are employed for photoanode sensitization and S/S^2−^ polysulfide solution is used as an electrolyte. Moreover, the front-side illuminated free-standing TNT arrays-based QDSSCs showed improved photovoltaic performance as compared to QDSSCs prepared using TiO_2_ particles or TNT on Ti substrate [51,52] or back-side illuminated cells [51,53]. The free-standing TNT arrays were detached from the Ti substrate and loaded with different types of QDs by sequential chemical bath deposition—successive ionic layer adsorption and reaction (i.e., SILAR). Up to date, the light-harvesting performance of free-standing TNT-array-based QDSSCs modified with CdS [36,51]; CdSe [52]; CdSe/CdS [53,56]; Zn_x_Cd_1-x_Se [54]; and CdS/CdTe [55] QDs have been reported. The best results of those studies were collated in Table 3.

Wang et al. [36] measured the highest energy conversion efficiency value of 3.34% for QDSSCs modified by CdS QDs. Li et al. [53] reported a power conversion efficiency of 2.57% for QDSSCs constructed using the free-standing TNT layer sensitized with CdSe/CdS QDs and covered by the ZnS layer using the SILAR deposition method. The ZnS coating improved the performance of the CdSe/CdS double-sensitized QDSSCs due to the formation of an energy barrier and passivation effect, protecting the QDs layer from polysulfide-caused corrosion. An improvement in the solar cell performance was also observed by Gualdrón-Reyes et al. [55] for the CdTe QDs-modified TNT array coated with the ZnS layer. Li et al. [54] used ternary Zn_x_Cd_1-x_Se QDs layers to modify the free-standing TNT layer in QDSSCs. The power conversion efficiency of QDSSCs increased with the increase of the Cd content, reaching 2.15%. It was proposed that the gradient structure of formed layers led to band position alignment (Fermi level alignment) and contributed to the improved performance of constructed QDSSCs.

**Table 3:** The best results of photovoltaic performance of QDSSCs, constructed using free-standing TNT arrays.

Anodizing Parameters	TNT Layer Detachment	TNT Layer Morphology			QDSSC Measurement							Ref.
		Thickness (μm)	Tube Diameter (nm)	Wall Thickness (nm)	Photoanode Preparation	Electrolyte	Active Area (cm^2^)	Results				
								Short- Circuit Current (mA cm^−2^)	Open-Circuit Voltage (V)	Fill Factor (%)	Conversion Efficiency (%)	
0.3 wt% NH_4_F, 3 wt% DI, in EG 1st: 40 V	- Annealing at 500 °C for 3 h - Anodizing at 15 V for 1 h - Self-detachment during 2nd anodization	12	Inner 100, outer 110	10	- Transferred onto FTO spin-coated with TiO_2_ layer and annealed at 450 °C and binded with a drop of 0.1 M Ti-isopropoxide in IPA - Annealed at 450 °C for 30 min - SILAR CdS QDs deposition (25 cycles)	0.5 M Na_2_S, 2.0 M S, 0.2 M KCl in H_2_O/methanol (3:7, vol/vol)	-	7.52	0.462	42	1.47	[51]
0.25 wt% NH_4_F, 1 M H_2_O, in EG 1st: 60 V, 3 h	- Annealing at 450 °C for 4 h - Anodizing at 12 V for 1 h - Amorphous layer chemical dissolution in 5% H_2_O_2_ for 2 h	18	80 ± 3	13	- Binded onto FTO with 0.1 M Ti-isopropoxide in IPA and 5 wt% ethyl cellulose - Annealed at 450 °C for 4 h - SILAR CdSe QDs deposition (8 cycles)	0.5 M Na_2_S, 2 M S, 0.2 M KCl, in H_2_O/methanol (3:7, vol/vol)	0.16	16.02	0.51	29.74	2.43	[52]
0.3 wt% NH_4_F, 3 vol% H_2_O, in EG 1st: 60 V, 8 h	- Annealing: 450 °C at 1 h - Anodizing at 10 V for 30 min - Rinsing in ethanol - Amorphous layer chemical dissolution in 10 wt% H_2_O_2_ for 6 h	5–6	Inner 90 outer 110	20	- Binded onto FTO with 2 drops of 2 mM tetrabutyl titanate in ethanol - Annealed at 450 °C for 30 min - SILAR CdSe (10 cycles) and CdS (8 cycles) QDs and ZnS (2 cycles) layer deposition	0.5 M Na_2_S, 0.1 M S, 0.01 M KCl, in H_2_O	0.25	13.27	0.44	44	2.57	[53]
0.3 wt% NH_4_F, 3 vol% H_2_O in EG 1st: 50 V, 8 h	- Annealing: 450 °C at 1 h - Anodizing at 10 V for 6 h - Amorphous layer chemical dissolution in 10 wt% H_2_O_2_ for 6 h	-	Outer 100 inner 80	~20	- Transferred onto FTO coated with tetrabutyl titanate, ethyl cellulose, terpinol and ethanol paste - Sintered at 450 °C for 30 min - SILAR Zn and Se QDs deposition - Substitution in Cd(NO)_3_ aqueous solution	0.5 M Na_2_S, 0.1 M S, in H_2_O	0.16	10.49	0.41	0.50	2.15	[54]
0.25 wt% NH_4_F, 2 wt% H_2_O, in EG 1st: 50 V, 3 h	- Annealing at 500 °C for 2 h - Amorphous layer chemical dissolution in 33 wt% H_2_O_2_ for 20 s *Barrier layer removal in 0.5 wt% oxalic acid	14.5	100	15	- SILAR deposition of CdS QDs (5 cycles) - Transferred to FTO doctor-bladed with TiO_2_ NPs paste and annealed at 450 °C for 30 min	0.5 M LiI, 0.05 MI_2_, 0.5 M tert-butylpyridine in anhydrous ACN	0.25	5.2	-	-	3.34	[36]
0.45 wt% NH_4_F, 2 wt% H_2_O, in EG 1st: 60 V, 2–3 h	- Annealing at 400 °C for 2 h - Anodizing at 60 V for 15 min - Amorphous layer chemical dissolution in 30 wt% H_2_O_2_ for 2 min	16.7–35.9	85.8–126.0	-	- Binded onto ITO with B-doped TiO_2_ sol - Calcinated at 400 °C - Immersion in CdTe QDs solution (4 h) - SILAR deposition of CdS QDs (8 cycles) - SILAR deposition of ZnS layers (2 cycles)	1.0 M Na_2_S, 1.0 M S	0.15	1.39	−0.29	30	0.16	[55]
0.3 wt% NH_4_F, 3 vol% H_2_O, in EG 1st: 60 V, 15 h	- Annealing at 450 °C for 2 h - Anodizing at 12 V for 6 h - Amorphous layer chemical dissolution in 10 wt% H_2_O_2_ for 12 h	20	100	-	- Binded to FTO glass with terpentiol, ethyl cellulose, ethanol and butyl titanate - Annealed at 450 °C for 1 h - SILAR deposition of CdS QDs (8 cycles) - SILAR deposition of CdSe QDs (9 cycles)	1.0 M Na_2_S, 1.0 M S, in H_2_O/methanol (1:1, vol/vol)	0.16	14.44	0.39	46	2.52	[56]

**Figure 7:**
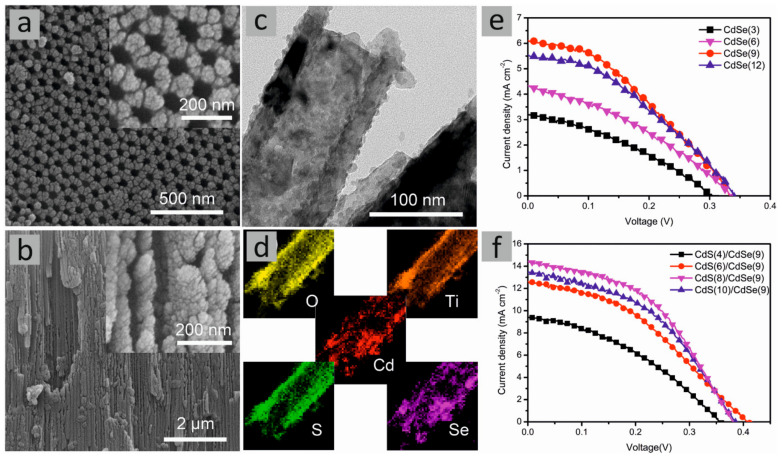
The (**a**) top, (**b**) cross-section SEM, and (**c**) TEM images, accompanied with (**d**) elemental mapping of CdSe and CdS QDs-modified TNT. The photovoltaic performance recorded for (**e**) CdSe QD TNT solar cells influenced by CdSe SILAR cycles and (**f**) CdSe(9)/CdS(x) TNT solar cells influenced by CdS SILAR cycles. Reprinted in part with permission from [56]. Copyright © 2018, Elsevier.

It was also observed that the increase in the number of SILAR cycles led to a higher amount of deposited QDS, improving the photovoltaic response of studied QDSSCs. However, at some point, further increasing the number of SILAR cycles caused a reduced energy conversion coefficient value and decreased *J_SC_* and *V_OC_*. This was explained by the excess of the generated QDs, enhancing recombination opportunity or their aggregation, blocking the nanotubes, and therefore hindering electrolyte infiltration [53,55]. Ren et al. [56] optimized the number of the SILAR cycles to deposit CdSe and CdS QDs, which led to an increased surface roughness of the TNT (Figure 7a,b). They significantly decreased the nanotube diameter, still providing space for electrolyte penetration. The TiO_2_ nanotubes were a scaffolding for CdSe and CdS QDs, which were uniformly distributed (Figure 7c,d). Notably, the CdS layer between the CdSe and TiO_2_ would facilitate the electron transfer between those two by realigning band edges and consequently lead to an improved CdSe QDs loading. As a result, the energy conversion coefficient of 2.52% was achieved (Figure 7e,f) [56].

### 3.6. Diffusion and/or Degradation of Organic Pollutants

Some of the organic pollutants, such as methylene blue (MB), acid orange 7 (AO7), methyl orange (MO), rhodamine B (RhB), pentachlorophenol (PCP), and others might be degraded relatively fast by photocatalysis. Promising results in toxic compounds degradation have been reported for using the self-organized and flow-through TNT membrane [137]. A significant advantage of the material is its photoactivity, size selectivity (effects of nanoporous structure), and ease of utilization in a static flow-through reactor. Albu et al. [37] conducted experiments to determine the permeability and photocatalytic activity of flow-through TNT membranes toward the decomposition of methylene blue. Spectrophotometric analysis confirmed that the organic pollutant decomposition occurred on the illuminated surface of the TNT membrane. The authors claimed that the diffusion rate, rather than the photocatalytic process rate, determined the rate of MB degradation. In addition, complete pollutant removal has been observed after a single flow-through cycle through the membrane. Passalacqua and co-workers [90] have reported convergent conclusions on the permeation of methylene blue molecules using a flow-through TNT membrane under dark conditions. The study was conducted on TiO_2_ nanotube membranes prepared by a multi-step anodizing process and a potential shock lift-off technique. The flow of methylene blue occurred through the channels of the membrane depending on the concentration gradient and continued until reaching equilibrium in the two-compartment reactor.

Further advancements in the flow-through TNT membrane application for diffusing and photodegrading organic pollutants have been made through improvements in material preparation [84]. Researchers have proposed fabricating defect-free TiO_2_ nanotube layers with high thermal stability through anodizing in a lactic acid electrolyte. Subsequent tube opening was achieved by dipping the TNT membrane in H_2_O_2_ aqueous solution, resulting in a free-standing form of TNT membrane. After annealing and under UV irradiation, the flow-through defect-free TNT membrane exhibited higher photocatalytic activity towards the degradation of acid orange 7 than the one with an amorphous structure. The convergent observation was made by Liao et al. [85] They found that the degradation of RhB increased due to the synergetic effect when the flow-through photocatalysis process was conducted over anatase TiO_2_ nanotube membranes. This means that an open-ended TNT membrane permits the degradation of RhB under simulated sunlight irradiation while diffusing through TiO_2_ nanotubes. Furthermore, the authors proposed a material modification that improved the activity to mitigate the adverse effects of environmental pollution. They fabricated CdS@TiO_2_ core-shell nanocables through a chemical reaction between Cd^2+^ and S^2−^ ions inside the flow-through TNT nanotubes at different reaction times. The CdS@TiO_2_ core-shell nanocables photocatalyst synthesized for 24 h was characterized by a rate constant (k) of 12 × 10^−4^ min^−1^, more than two times higher than the bare TNT nanotubes (k = 5 × 10^−4^ min^−1^) [85]. In general, enhanced photocatalytic activity was attributed to heterojunction formation, which improved the separation of photogenerated charge carriers and extended the absorption range into visible light. In addition, the formation of the CdS@TiO_2_ core-shell framework did not limit RhB diffusion. Subsequent works have proven that not only does the formation of composite photocatalysts play a role in pollutant degradation, but also an annealing temperature that influences the crystal structure of titania. Pan et al. [86] investigated the impact of annealing temperature on the photocatalytic activity of CdS/TiO_2_ nanotube arrays (TNTa) in methyl orange degradation. The annealing temperature of the composite photocatalyst ranged from 300 °C to 600 °C. The highest degradation efficiency of MO was achieved when the CdS/TNTa were annealed at 600 °C, resulting in 62% pollutant removal. In contrast, only 25% of the pollutant was removed when the photocatalyst was annealed at 300 °C. Computational results have also confirmed the photocatalytic performance of the CdS/TNTa catalyst, indicating 3.6 times higher reaction rate for the sample annealed at 600 °C (k = 6.85 × 10^−3^ min^−1^) than for unmodified TNTa (k = 1.88 × 10^−3^ min^−1^). These results are consistent with previous reports and point out the crucial role of crystallographic transformation induced by higher temperatures [87,88,138]. Further improvement in photogenerated charge carrier separation during the breakdown of organic pollutants has been obtained by introducing metal nanoparticles [89]. A highly oriented TNT membrane was uniformly decorated with Ag nanoparticles through a wetting thermal decomposition for efficient photocatalytic degradation of methylene blue and pentachlorophenol. The metal nanoparticles acted as electron trapping centers, effectively suppressing the recombination of photogenerated electrons and holes while improving the system’s photocatalytic activity.

The prepared self-organized and flow-through TNT membranes applied towards diffusion and photodegradation of organic pollutants were characterized using XRD, SEM/TEM/EDX mapping to provide information about crystal phase, morphology and nanotubes dimensions, and elemental composition [85,86,89]. DRS measurements have been utilized to investigate the optical properties of the materials and evaluate the band gap value of proposed CdS/TiO_2_ nanotube arrangements [86]. Additionally, the authors [86,89] discussed the charge transfer and conductivity within the system based on electrochemical impedance spectroscopy, cyclic voltammetry, and current-voltage measurements due to the modification with noble metal nanoparticles.

### 3.7. Biomedical Application (Biofiltration—Sensor)

Self-organized TNT membranes are found to be useful in biofiltration and/or sensing applications due to biocompatibility, unique and controllable nanotube architecture, thermal stability, corrosion resistance, and semiconducting behavior. Paulose and co-workers [91] have studied how model molecules, such as glucose and immunoglobulin G (IgG), diffuse through the polycrystalline TiO_2_ nanotube membranes. These membranes have uniform nanopores distribution with a size of 125 ± 10 nm and a nanotube length of 200 µm. The authors observed a linear relationship between molecular weight (MW_glucose_ = 180 Da; MW_IgG_ = 150 kDa) and the natural logarithm of the diffusion coefficient (D) through the membrane, which was 2.42 × 10^−4^ min^−1^ and 1.73 × 10^−7^ min^−1^ for glucose and IgG, respectively. This result indicates a zero-order diffusion system, where concentration does not affect the rate. Consequently, the separation of molecules was based on pore size [91]. Similar observation has also been done when the amorphous TiO_2_ nanotube membrane (length 60 µm; 110 and 75 nm inner tube diameter) was applied for biofiltration. Obtained diffusion rates were correlated with the molar mass and size of utilized standard molecules (38.2 nmol h^−1^ for glucose, 0.03 nmol h^−1^ for insulin, and 0.0002 nmol h^−1^ for IgG) [92].

Moreover, self-organized TiO_2_ nanotubes have been found to be effective in improving the detection level of fluorescence-labeled proteins in an immunoassay concept. In a study by Song et al. [94], TiO_2_ nanotube arrays were used as a substrate for anti-rabbit (Ab) immunoglobulin G (IgG), which further had been utilized for selective coupling with rabbit IgG. The authors reported a 100-fold increase in the detection limit (0.01 pg mL^−1^; TNTs 18 µm thick and 75 ± 5 nm diameter) when compared to quantum-dot immunosensors proposed by Cui et al. [93] In addition, the authors [94] drew attention to the self-cleaning ability resulting from the photocatalytic properties of polycrystalline TiO_2_ nanotubes. The features of anodic TiO_2_ nanotube membranes toward selective separation of biomolecules can be further improved by tailoring surface properties, such as wettability. Xu et al. [50] reported on enhanced loading capacity and improved protein selectivity when TNT membrane was decorated with graphitic carbon patches (C/TNT). The saturated adsorption capacity of human serum albumin (HAS) for the C/TNT membrane (41.8 mg g^−1^) was around 2.5 times higher than for the bare TNT membrane (~16.7 mg g^−1^) under the same experimental conditions. Moreover, the authors indicated a negligible difference in the surface area of analyzed materials, thus attributing improved protein extraction to the diverse surface properties of the tubes. Additionally, the reported results have shown that adjusting the pH of the solution could alter the adsorption behavior, thus enhancing the selectivity of the C/TNT membrane. After conducting stability tests, when subjected to UV-cleaning, the C/TNT sample maintained around 85% of its initial adsorption efficiency in each of the five subsequent cycles [50].

Bayram and co-workers [95] studied the electrostatic repulsive features of anodic TiO_2_ nanotube-based membranes with diverse surface nanomorphology in cross-flow biofiltration applications. In particular, creatinine clearances and bovine serum albumin (BSA) rejection were analyzed. The free-standing TNT membranes were formed by multi-step anodizing followed by lift-off with a sudden voltage increase at the end of the second anodizing step. By adjusting detachment conditions (i.e., electrolyte solution, temperature or time) the researchers were able to tune the morphology of the fabricated free-standing TiO_2_ nanotube-based membrane. Among the three types of prepared samples, specifically aligned TNT with closed-ends, aligned TNT with opened-ends, and chaotic nanowire membranes the best results in terms of creatinine clearance (97.25 ± 3.28%) and BSA rejection (18.33 ± 2.53%) were obtained with the middle one. The open-ended TNT membrane was characterized by the highest hydrophilicity and thus the best mechanical stability during the cross-flow of blood-mimicking solution. The researchers attributed the antifouling feature of the membrane to the electrostatic repulsive force between the negatively charged hexafluorotitanate (TiF_6_^2–^)-rich region and BSA at physiological alkalinity (pH 7.5). However, they noted that further improvement in charged protein rejection is necessary for real-life biomedical applications despite the promising reported results.

Beyond the previously mentioned characterization methods, such as SEM and XRD, which provide information about the morphology, nanotube dimensions, and chemical composition of the material, Song et al. have utilized XPS analysis to confirm the surface changes of titanium nanotubes after immobilization of immunoglobulin G [94]. The modification that occurred on the self-organized TNT membranes when applied to biofiltration, specifically the investigation of functional groups attached to the membrane, might be identified by Fourier transform infrared spectroscopy (FT-IR) [50]. In addition, Xu et al. [50] have performed XRD, Raman spectroscopy, and XPS measurements to confirm the presence and nature of carbon over the TNT membrane.

### 3.8. Micromotors

An innovative and interesting application of free-standing flow-through TiO_2_ nanotube membranes is to use them as micro/nano-motors. These micro-/nano-objects can convert chemical energy into kinetic energy, allowing them to move independently within fluids. As a result, they can be used in various applications, including cargo delivery, protein and cell separation, water remediation, and microsurgery. Wang et al. [96] have demonstrated UV light-controlled two-dimensional amorphous TiO_2_ plate micromotors that displayed autonomous motion in a hydrogen peroxide solution without any surfactants. The morphology, elemental composition and amorphous nature of utilized free-standing flow-through TiO_2_ nanotube membranes have been verified by commonly available methods, specifically SEM/EDX and XRD. According to the authors of the study, movements of amorphous TiO_2_ 2D micromotors are directly related to photogenerated oxygen bubbles and might be controlled by exposure to UV light. Eliminating surfactants, commonly used to lower interfacial tension and bubble detachment from micro/nano-motors, encourages biological applications. This direction of free-standing flow-through TiO_2_ nanotube membranes application seems to be interesting. However, further analysis for common biological use of the micro/nano-motors is crucial if only because of their activity in more complex fluid electrolytes.

### 3.9. Electrochromic Devices

Anodic self-organized TiO_2_ nanotube membranes possess interesting electrochromic properties, making them a promising material for optical devices. When exposed to external bias and subsequent redox reactions, titania has the unique ability to reversibly change color, altering its electronic structure and optical properties. TiO_2_ nanotubes formed anodically and attached to transparent conducting glass have shown better electrochromic features in terms of H^+^ intercalation from 0.1 M HClO_4_ aqueous solution than the layer of nanoparticles (commercially available Degussa P25), even for much thinner layers of TNTs [97]. When attached to conducting glass, the TNT membrane exhibits higher transmittance, optical contrast, and cycling stability compared to immobilized nanoparticle layers. The authors attributed the benefits of using TiO_2_ nanotubes to the much narrower diffusion path and noticeably lower resistivity losses in the tube wall structure than the nanoparticle one.

Another interesting approach to achieving large-scale, flexible nanotubular flow-through TNT membrane with improved electrochromic properties has been proposed by Albu, Schmuki and co-workers [98]. The TNT membrane was made using a double-layer structure of titanium and aluminum. The authors utilized photolithographic patterning of the electrode to create squares of anodizing areas separated by metallic titanium. The best results were achieved with patterned TiO_2_ membranes with the smallest grits of 85 × 85 μm^2^, producing the highest switching rate (k = 8.9 ms^−1^) and transmission during Li^+^ ion intercalation. Additionally, filtration experiments proved that the proposed fabrication method produced an open-through membrane with uniform filtration properties, thus expanding the potential range of applications [98].

Hwang et al. [99] proposed the use of dye-sensitized flow-through anodic TiO_2_ nanotube membranes as optically controlled gates in nanofluidic. In this work, the TNT surface was decorated with N719 Ru(II) dye ([Ru^II^(2,2′-bipyridyl-4,4′-dicarboxylate)-(NCS)_2_]TBA_2_; TBA—tetra-n-butylammonium), commonly utilized in DSSC. The authors claimed that under visible light illumination, the N719 dye/TNT interface’s electric properties might be modified, affecting the hydrodynamic properties and thus the permeability of the TiO_2_ nanotubes. Specifically, under specific light illumination, the excited electrons from the LUMO of the dye are injected into the CB of the titania. This leads to the forming of a negatively charged depletion layer near the surface of TiO_2_ and a positively charged layer of adsorbed dye molecules, which affects the movement of free dye anion in the solution. A similar effect has been reported during the transportation of polystyrene nanospheres through the N719 dye/TNT. This visible-light-promoted effect was reversibly modulated by switching on and off the light. It can be considered an additional factor for further material application in diverse fluidic devices.

The morphology and crystallographic properties of prepared anodic self-organized TiO_2_ nanotube membranes have been analyzed by SEM and XRD methods [97,98,99]. The elemental composition has been evaluated by XPS analysis [99]. In addition, Ghicov et al. [97] have performed stability measurements by utilizing CV and chronoamperometric methods. The optical performance of the samples has also been analyzed by detailed optical and spectrochemical measurements [97,98,99].

## 4. Conclusions and Future Outlook

This paper reviews current approaches for achieving free-standing anodic TiO_2_ nanotube arrays, characterized by well-ordered structure and large surface area. Firstly, a brief summary of the suitable anodizing conditions and mechanism behind the TNTs formation has been done according to the currently available literature. Secondly, several methods for detaching free-standing TiO_2_ nanotube arrays from Ti substrate and forming flow-through TNT membranes reported in the literature have been identified and discussed in detail. Among them, mechanical, chemical, and physical parameters mediated detachment along with dry etching by inductively coupled plasma might be distinguished. Moreover, chemical etching by hydrofluoric vapor, dilute hydrofluoric acid/sulfuric acid solution, and oxalic acid are effective methods for opening the bottom of the TiO_2_ nanotube membranes.

The data collected in Table 1, Table 2 and Table 3 demonstrate that the anodizing conditions of Ti substrate, particularly electrolyte composition, time, and number of steps determine the thickness/length and diameter of nanotubes. Both temperature and time during annealing have a certain impact on the structure and photocatalytic properties of fabricated TiO_2_ nanotube membranes. The utilized detachment method and applied conditions affect whether the obtained TNT membrane is free-standing and one or both sides open. Diverse posttreatment strategies such as composite formation have been overviewed according to different membrane applications. Additionally, the way the membrane is immobilized on a conducting substrate or installed in an experimental cell/reactor is crucial and varies in the cited research articles. All these factors are significant for the resulting TNT membranes, making it non-trivial to compare the presented results. Thus, all of these parameters should be considered for future experiments over TNT arrays/membranes, regardless of their application.

The free-standing/flow-through membranes possess unique (photo)catalytic properties, which make them highly suitable for use in energy, environmental, biomedical, and/or sensing applications. This work highlights the current status and prospects of these membranes in fields such as hydrogen generation, fuel and solar cells, Li-S batteries, diffusion and degradation of organic pollutant, membrane filters, micromotors, and electrochromic devices. Despite scientific efforts, preparing large-scale free-standing, flow-through TNT membranes, enabling future commercial application, remains a challenge. Therefore, it is necessary to conduct further research to understand the formation mechanism and functional properties of TNT membranes. This understanding will enable the integration of TNT membranes into real-world devices. Furthermore, advancing our knowledge of TiO_2_ nanotube growing mechanisms is also essential for environmental reasons. The above is a solid foundation for proposing an alternative to the toxic, fluoride-based electrolytes commonly utilized during anodizing. In addition, the presence of the fluoride-rich layer affects the nanotube layer adhesion to the substrate when bending. Thus, adhesion is another aspect that should be considered when preparing TNT membrane. Additionally, enhancing optical properties and reducing charge carrier recombination are constantly required to effectively and profitably utilize free-standing, flow-through TNT membranes in a light-induced reaction. Thus, optimizing material doping and modification approaches should be further addressed through complementary experimental and theoretical studies.

## Data Availability

No new data were created or analyzed in this study.
